# Parallel Evolution of X Chromosome-Specific Structural Maintenance of Chromosomes Complexes in Two Nematode Lineages

**DOI:** 10.1093/molbev/msaf270

**Published:** 2025-10-24

**Authors:** Avrami Aharonoff, Jun Kim, Aaliyah Washington, Sevinç Ercan

**Affiliations:** Department of Biology, Center for Genomics and Systems Biology, New York University, New York, NY 10003, USA; Department of Biology, Center for Genomics and Systems Biology, New York University, New York, NY 10003, USA; Department of Biology, Center for Genomics and Systems Biology, New York University, New York, NY 10003, USA; Department of Biology, Center for Genomics and Systems Biology, New York University, New York, NY 10003, USA

**Keywords:** dosage compensation, sex chromosomes, X chromosome, nematodes, *Steinernema*, *Pristionchus*, *Caenorhabditis*, *Oscheius*, Hi-C, TAD, condensin, structural maintenance of chromosomes, SMC complexes, evolution, parallel evolution, transcription, H4K20me1

## Abstract

Mechanisms of X chromosome dosage compensation have been studied in model organisms with distinct sex chromosome ancestry. However, the diversity of mechanisms as a function of sex chromosome evolution is largely unknown. Here, we anchor ourselves to the nematode *Caenorhabditis elegans*, where dosage compensation is accomplished by an X chromosome–specific condensin that belongs to the family of structural maintenance of chromosomes (SMC) complexes. By combining a phylogenetic analyses of the *C. elegans* dosage compensation complex with a comparative analysis of its epigenetic signatures, such as X-specific topologically associating domains and enrichment of H4K20me1, we show that the condensin-mediated mechanism evolved recently in the lineage leading to *Caenorhabditis* following an SMC-4 duplication. Unexpectedly, we found an independent duplication of SMC-4 in *Pristionchus pacificus* along with X-specific topologically associating domains and H4K20me1 enrichment, which suggests that condensin-mediated dosage compensation evolved more than once in nematodes. Differential expression analysis between sexes in several nematode species indicates that dosage compensation itself precedes the evolution of X-specific condensins. In Rhabditina, X-specific condensins may have evolved in the presence of an existing mechanism linked to H4K20 methylation as *Oscheius tipulae* X chromosomes are enriched for H4K20me1 without SMC-4 duplication or topologically associating domains. In contrast, *Steinernema hermaphroditum* lacks H4K20me1 enrichment, SMC-4 duplication, and topologically associating domains. Together, our results indicate that dosage compensation mechanisms continue to evolve in species with shared X chromosome ancestry, and SMC complexes may have been co-opted at least twice in nematodes, suggesting that the process of evolving chromosome-wide gene regulatory mechanisms are constrained.

## Introduction

According to the classic model of sex chromosome evolution, X and Y chromosomes evolved from a homologous pair of autosomes that acquired a sex determining gene (Charlesworth and Charlesworth 1978; Jablonka and Lamb 1990; Charlesworth 1991, 1996; Hodgkin 1992). The degree of heteromorphy between X and Y chromosomes differs among taxa (Furman et al. 2020). In some taxa, X and Y chromosomes remain homomorphic and continue to recombine (Wright et al. 2016; Furman et al. 2020). In others, suppression of recombination between the X and Y chromosomes leads to the degeneration of the Y and almost complete hemizygosity of the male X chromosome (Charlesworth and Charlesworth 1978; Rice 1987; Bachtrog 2013). In extreme cases like nematodes, the Y chromosome was lost entirely. The loss of genes on the Y chromosome necessitated mechanisms to maintain ancestral gene dose in dosage-sensitive gene networks with a mix of autosomal and sex chromosomal genes (Oliver 2007; Lenormand and Roze 2025). These genetic and epigenetic mechanisms compensating for the imbalance in gene dose between males and females are collectively referred to as “dosage compensation” (Charlesworth 1996).

**Fig. 1.**
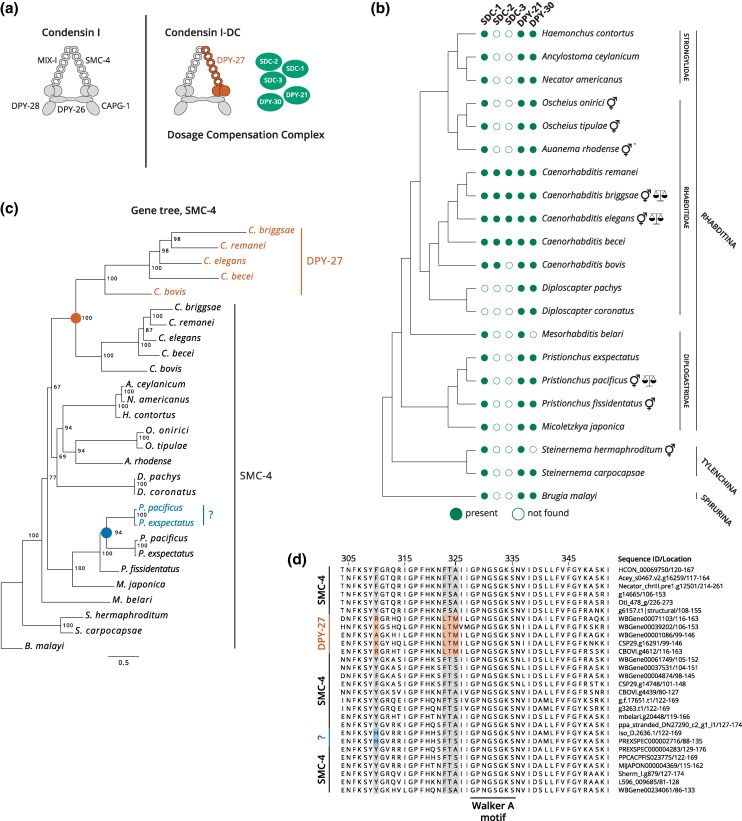
Independent duplications of SMC-4 in *Caenorhabditis* and *Pristionchus* generated novel SMC-4 proteins. a) Condensin I-DC and five noncondensin proteins (green) make the *C. elegans* DCC. Condensin I-DC shares all but one subunit, DPY-27, with the canonical condensin I. b) Proteomes with BUSCO scores >85% were run in OrthoFinder and grouped as orthogroups. SDC-2 and SDC-3 only appear in *Caenorhabditis*, whereas SDC-1, DPY-21, and DPY-30 are found in all three clades. *, trioecious; 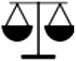, known chromosome-wide dosage compensation. c) OrthoFinder results were used to generate a maximum-likelihood tree of SMC-4. DPY-27 is an SMC-4 paralog that arose in the lineage leading to *Caenorhabditis* (orange circle). An independent duplication of SMC-4 occurred in *Pristionchus* (blue circle). To make the SMC*-*4 gene tree more readable, species names were substituted for gene names. Gene names can be found in Table 2. Node values represent bootstrap scores. The scale bar represents branch length. d) Multiple sequence alignment of the N-terminal ATPase domain highlights conserved and distinct amino acid sequences in DPY-27 and SMC-4. Within this region, example amino acid changes that support the duplication and divergence of DPY-27 in *Caenorhabditis* are shown in orange, and changes that support the independent duplication of SMC-4 in *Caenorhabditis* and *Pristionchus* are shown in blue. Tick marked numbers reflect coordinate in the multiple sequence alignment. Numbers by gene names reflect coordinate in sequence.

Sex chromosomes have coevolved with dosage compensation many times. Therefore, clades defined by a shared ancestral X chromosome can also be defined by their dosage compensation mechanism. For example, mammals, flies, and nematodes all use different strategies. In *Mus musculus* (mouse), a long noncoding RNA, *Xist*, initiates several heterochromatic pathways to randomly inactivate one of the X chromosomes in females (XX) (Sahakyan et al. 2018; Loda and Heard 2019; Brockdorff et al. 2020). In *Drosophila melanogaster* (fly), a histone acetylating ribonucleoprotein complex, “male specific lethal” (MSL) activates transcription by about 2-fold on the single X chromosome in males (XY) (Straub et al. 2005; Lucchesi and Kuroda 2015). Finally, in the nematode *Caenorhabditis elegans*, the “dosage compensation complex” (DCC) is driven by an X-specific structural maintenance of chromosomes (SMC) complex that represses transcription by about 2-fold in hermaphrodites (XX) (Meyer and Casson 1986; Chuang et al. 1994; Csankovszki et al. 2009; Ercan 2015; Albritton and Ercan 2018) (Fig. 1a).

The formation of heteromorphic X and Y chromosomes is *not* an end point. Chromosomal duplications, deletions, fusions, and translocations continue to change sex chromosomes (Landeen and Presgraves 2013; Bachtrog et al. 2014; Myosho et al. 2015; Vicoso and Bachtrog 2015; Furman et al. 2020). Therefore, although initial dosage compensation mechanisms evolve in response to Y chromosome degradation, they continue to evolve in response to X chromosome evolution. For example, in two species of *Drosophila*, a translocation of autosomal sequences to the X chromosome selected for DNA sequence motifs capable of recruiting the MSL complex to the neo-X region (Bone and Kuroda 1996; Ellison and Bachtrog 2013, 2019). While the motifs required for MSL recruitment remained similar in the *Drosophila* system, in two *Caenorhabditis* species, *C. elegans* and *C. briggsae* (estimated to have diverged from *C. elegans* 15 to 30 Ma), the DNA sequence motifs that recruit the DCC have functionally diverged (Yang et al. 2023).

The studies performed in *Drosophila* used species in which MSL complex subunits were readily identified by sequence homology (Bone and Kuroda 1996; Ellison and Bachtrog 2013, 2019). Similarly, subunits of the *C. elegans* DCC were identified by sequence homology in *C. briggsae* (Yang et al. 2023). Studying the mechanism of dosage compensation in more distantly related species, however, is difficult due to the rapid evolution of proteins involved in sex determination and dosage compensation (Hodgkin 1992; Bachtrog et al. 2014). Furthermore, until recently, few nonmodel organisms had chromosome scale assemblies. Therefore, the field remains largely ignorant to the diversity in dosage compensation mechanisms in species that have diverged significantly, but share an X chromosome ancestor.

Here, we used a combination of phylogenetic and genomic approaches to explore dosage compensation in several species of nematodes with significant differences in X chromosome content. We anchored our work in *C. elegans*, a model organism in which XX and XO animals develop as hermaphrodites and males, respectively (Albritton and Ercan 2018). The core of the *C. elegans* DCC is a condensin complex belonging to the SMC family (Strunnikov and Jessberger 1999; Hagstrom et al. 2002; Harvey et al. 2002; Hirano 2005; Csankovszki et al. 2009). Most animals harbor two canonical condensins (I and II) that mediate DNA loops to compact chromosomes and are essential for chromosome segregation in mitosis and meiosis (Hirano 2005). Condensins I and II share a heterodimer of SMC-2 and SMC-4 subunits, which interact with a different set of three chromosome-associated proteins (Hirano 2012). In *C. elegans*, the third condensin (I^DC^) is formed by replacing SMC-4 with DPY-27 in condensin I (Fig. 1a) (Csankovszki et al. 2009). Condensin I^DC^ (hereafter I-DC) interacts with five noncondensin proteins to form the DCC (Csankovszki 2009).

The DCC mediates two major epigenetic changes on the X chromosomes: (i) the formation of loop-anchored topologically associating domains (TADs) and (ii) the enrichment of H4K20me1 in females and hermaphrodites (Vielle et al. 2012; Kramer et al. 2015; Anderson et al. 2019; Kim et al. 2022). To explore dosage compensation across distantly related nematodes, we first generated a phylogeny of SMC-4 and DPY-27 in Rhabditina, Tylenchina, and Spirurina. We then looked for condensin-mediated epigenetic phenotypes by performing Hi-C and ChIP-seq in several species. Based on the presence of two SMC-4 paralogs, X-specific loop-anchored TADs, and X enrichment of H4K20me1, we conclude that condensin-mediated dosage compensation arose more than once, the other time being in *Pristionchus.* In *Oscheius*, we found evidence for dosage compensation and upregulation of H4K20me1, but not for TADs, and in *Steinernema*, we found an absence of dosage compensation, TADs, and H4K20me1. We surmise that dosage compensation is ever changing in nematodes, and that the mechanism in the common ancestor of *Oscheius, Caenorhabditis*, and *Pristionchus* may be through the well conserved histone H4 Lysine 20 demethylase DPY-21. We propose that the X-specific condensin arose in the lineage leading to *Caenorhabditis* in the presence of this mechanism. Our findings highlight the continued and constrained evolution of dosage compensation mechanisms and a previously underappreciated diversity of strategies in species with a shared X chromosome ancestry.

## Results

### Ortholog Analysis Reveals Variable Levels of Conservation Among DCC Subunits

Nematodes represent a large number of species with distinct genomes. We used a recent phylogenomic analysis to choose a set of species from Rhabditina (clade V) and Tylenchina (clade IV) (Fig. 1b) (Ahmed et al. 2022). All species selected had BUSCO scores of at least 85% with the exception of *Steinernema carpocapsae*, which fell at 76% (Table 1). In all, we sampled from the Strongylidae, Rhabditidae, Dipoglastridae, and Steinernematidae families, which display significant divergence in sex chromosome content (Fig. S1) (Tandonnet et al. 2019; Foster et al. 2020; Gonzalez de la Rosa et al. 2021). Our outgroup was *Brugia malayi* (Spirurina, clade III). To determine the conservation of the DCC, we searched for the orthologs with OrthoFinder, which uses a reciprocal best blast hits approach (Emms and Kelly 2015; Emms and Kelly 2019).

**Table 1.** Species sampled for phylogenetic analysis of dosage compensation proteins

Species	Clade	Family	BUSCO (S/D/F/M)
*H. contortus*	Rhabditina	Strongylidae	85/11/1/3
*Ancylostoma ceylanicum*	Rhabditina	Strongylidae	86/9/2/3
*Necator americanus*	Rhabditina	Strongylidae	46/51/1/2
*O. onirici*	Rhabditina	Rhabditidae	89/3/2/6
*O. tipulae*	Rhabditina	Rhabditidae	88/3/2/8
*Auanema rhodense*	Rhabditina	Rhabditidae	90/1/1/8
*C. remanei*	Rhabditina	Rhabditidae	95/4/1/0
*C. briggsae*	Rhabditina	Rhabditidae	80/19/0/0
*C. elegans*	Rhabditina	Rhabditidae	66/34/0/0
*C. becei*	Rhabditina	Rhabditidae	89/9/1/2
*C. bovis*	Rhabditina	Rhabditidae	77/17/1/6
*Diploscapter pachys*	Rhabditina	Rhabditidae	​89/2/2/8
*Diploscapter coronatus*	Rhabditina	Rhabditidae	89/2/2/8
*Mesorhabditis belari*	Rhabditina	Rhabditidae	72/13/2/13
*Pristionchus exspectatus*	Rhabditina	Diplogastridae	90/3/4/4
*P. pacificus*	Rhabditina	Diplogastridae	96/2/2/0
*Pristionchus fissidentatus*	Rhabditina	Diplogastridae	89/4/5/2
*M. japonica*	Rhabditina	Diplogastridae	88/2/5/5
*S. hermaphroditum*	Tylenchina	Steinernematidae	43/45/1/11
*S. carpocapsae*	Tylenchina	Steinernematidae	58/18/4/21
*B. malayi*	Spirurina	Onchocercidae	68/31/0/1

S, complete and single copy; D, complete and duplicated; F, fragmented; M, missing. Completeness is measured by summing S and D.

The DCC subunits sex determination and dosage compensation-1 (SDC-1), SDC-2, and SDC-3 function in both sex determination and dosage compensation (Chu et al. 2002). It is well documented that sex determination mechanisms evolve with relative rapidity and are highly flexible to change (Hodgkin 2002; Bachtrog et al. 2014). SDC-2 is expressed specifically in XX embryos, and initiates hermaphrodite development and X chromosome dosage compensation (Dawes et al. 1999). SDC-3 physically interacts with SDC-2, helping repress the male determination gene *her-1* and recruit the DCC to the X chromosomes (Chu et al. 2002). Consistent with their role as upstream regulators of sex determination, we only find orthologs of SDC-2 and SDC-3 in *Caenorhabditis* (Fig. 1b).

Unlike SDC-2 and SDC-3, SDC-1 is not an essential gene, and is not required for DCC binding to the X chromosomes (Chuang et al. 1996), but contributes to *her-1* repression (Villeneuve and Meyer 1987, 1990; Chu et al. 2002; Meyer 2022). We found SDC-1 orthologs in all except for *Diploscapter* (Figs. 1b and S2). In *Caenorhabditis bovis, Caenorhabditis becei, C. elegans, C. briggsae, Caenorhabditis remanei, Micoletzkya japonica, Steinernema hermaphroditum*, and *S. carpocapsae*, multiple orthologs were identified (Fig. S2). SDC-1 is a predicted DNA binding transcription factor (TF) with seven zinc fingers (Nonet and Meyer 1991). It is possible that the conservation and expansion of SDC-1 is related to its TF function in hermaphrodite differentiation.

The DPY-21 subunit of the DCC contains clear orthologs across all species analyzed, and DPY-30 in all but two (Fig. 1b). DPY-21 is a conserved histone demethylase that is expressed in both the germline and soma with functions outside dosage compensation (Brejc et al. 2017). DPY-30 is a subunit of both the DCC and COMPASS, a chromatin modifying complex that is conserved across species (Pferdehirt et al. 2011; Ernst and Vakoc 2012; Morgan and Shilatifard 2020). Within COMPASS, DPY-30 interacts with ASH-2 and is required for trimethylation of H3K4, which is associated with active gene promoters (Simonet et al. 2007; Pferdehirt et al. 2011; Beurton et al. 2019). The nondosage compensation roles of DPY-21 and DPY-30 in chromatin regulation may contribute to their deeper conservation.

### Phylogenetic Analysis of SMC-4 Reveals Two Independent Duplications in *Caenorhabditis* and *Pristionchus*

In condensin I-DC, DPY-27 replaces SMC-4, one of the two ATPase subunits of the canonical condensin I. To determine whether DPY-27 is a paralog of SMC-4 and to uncover the timing of the proposed duplication, we identified SMC-4 homologs from the OrthoFinder results and produced a maximum-likelihood tree of the sequences (Fig. 1c; Table 2). Our tree suggests that DPY-27 is an SMC-4 paralog and is at least as old as the common ancestor of *C. elegans* and *C. bovis*, but younger than the common ancestor shared between *Caenorhabditis* and *Diploscapter* (Fig. 1c).

**Table 2.** Homologs of *C. elegans* SMC-4

Species	Homologs		
*H. contortus*	HCON_00069750		
*A. ceylanicum*	Acey_s0467.v2.g16259		
*N. americanus*	Necator_chrIII.pre1.g12501		
*O. onirici*	g14665		
*O. tipulae*	Oti_478_*g*		
*A. rhodense*	g6157.t1		
*C. remanei*	CRE20351^a^	CRE24237	CRE15073^b^
*C. briggsae*	CBG18034	CBG20143	
*C. elegans*	F35G12.8	R13G10.1	
*C. becei*	CSP29.g14748	CSP29.g16291	
*C. bovis*	CBOVI.g4439	CBOVI.g4612	
*D. pachys*	g.f.17651.t1		
*D. coronatus*	g3263.t1		
*M. belari*	mbelari.g20448		
*P. exspectatus*	PREXSPEC000002716	PREXSPEC000004283	
	PREXSPEC000022998^b^		
*P. pacificus*	ppa_stranded_DN27290_c2_g1_i1	Iso_D.2636.1	
*P. fissidentatus*	PPCACPFIS023775		
*M. japonica*	MIJAPON000004369^c^		
*S. hermaphroditum*	Sherm_I.g879		
*S. carpocapsae*	L596_009685		
*B. malayi*	WBGene00234061		

^a^CRE20350 was also called an ortholog. Upon inspection, it is misannotated and is in fact the three prime end of CRE20351 (SMC-4). Only CRE20351 was used in downstream analyses.

^b^These orthologs did not meet the sequence cover threshold of 50% and were not used in downstream analyses.

^c^MIJAPON000005949 was also called an ortholog. Upon inspection, both are segments of the same gene, and their separation is a result of misannotation. Only MIJAPON000004369 was used in downstream analyses.

Interestingly, we observed an independent duplication of SMC-4 in the *Pristionchus* lineage. To examine the paralogs, we performed a multiple sequence alignment of the N- and C-terminal ATPase domains, as well as in regions that were previously found to be conserved and essential for SMC-4 ATPase function (Hirano and Hirano 2006). Similar to other SMC subunits, the ATPase activity of DPY-27 is essential for its function (Breimann et al. 2022). *C. elegans* DPY-27 has the conserved Walker A motif on the N terminus (Harvey et al. 2002), along with site specific changes to the ancestral SMC-4 sequence (Fig. 1d and S3). In *Pristionchus pacificus* and *Pristionchus exspectatus*, the Walker A motif is also conserved in both paralogs. Furthermore, we observe site specific changes independent of *Caenorhabditis* upstream of the Walker A motif (Fig. 1d). We noticed that one group of paralogs in *P. pacificus* and *P. exspectatus* contains conserved changes to the ancestral SMC-4 sequence, suggesting that Iso_D.2636.1 and PREXSPEC000002716 diverged for a new function.

### Hi-C Analysis Reveals X-Specific TADs in *P. pacificus*

**Fig. 2.**
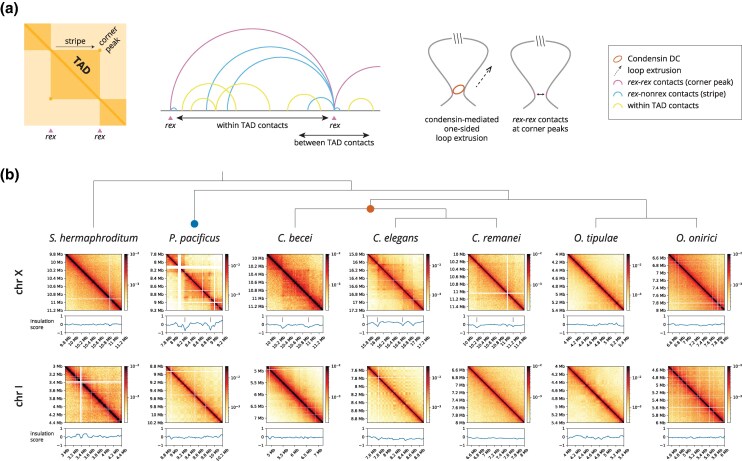
X chromosome–specific TADs are present in *P. pacificus*, but are absent in *S. hermaphroditum*, *O. tipulae*, *and O. onirici*. a) Schematic diagram of loop-anchored TADs on the *C. elegans* X chromosome (left). Features are annotated. DNA contacts are higher within TADs than between (middle left). *C. elegans* TAD boundaries are formed by strong *rex* sites, reminiscent of mammalian CTCF binding elements, and are proposed to function by loading and blocking one-sided DNA loop extrusion of condensin I-DC (middle right). b) Hi-C matrices of representative X and autosome windows at 5 kb resolution with insulation scores below (150 kb window). *X* axis (chromosome coordinate) is shared between the Hi-C matrix and the insulation score plot. The *Y* axis of the Hi-C matrix also represents chromosome coordinates. The color bar reflects relative interaction between a pair of bins, shown on a logarithmic scale. Empty bins in the Hi-C matrix (white) result in insulation score dips and are discarded for consideration as boundaries. The *P. pacificus* X chromosome displays loop-anchored TADs reminiscent of *C. elegans* dosage compensation along with dips in insulation score at TAD boundaries (tick marks). *S. hermaphroditum*, *O. tipulae*, and *O. onirici* do not display X chromosome–specific TADs. Blue and orange circles are placed on the tree where SMC-4 duplications occurred in *Pristionchus* and *Caenorhabditis*, respectively.

In *C. elegans*, condensin I-DC-mediated DNA loop extrusion increases 3D DNA contacts, and forms loop-anchored TADs specifically on the X chromosomes (Anderson et al. 2019; Kim et al. 2022). A small number of cis-regulatory elements that recruit the DCC to the X chromosomes (recruitment elements on the X, *rex*) form the boundaries between TADs and contact each other over hundreds of kilobase distances, forming *rex–rex* loops (Anderson et al. 2019; Kim et al. 2022) (Fig. 2a).

An independent paralog of SMC-4 is found in *P. pacificus* and *P. exspectatus* (Fig. 1c). If these paralogs function in dosage compensation, we would expect to find loop-anchored TADs on the X chromosomes of these species. In addition, we would not expect to find loop-anchored TADs in species without an SMC-4 paralog. To test our predictions, we compared the Hi-C features of the X chromosomes to that of autosomes in six of our sampled species (Fig. 2). We generated new data in *P. pacificus* and *Oscheius tipulae* and obtained previously published data in *S. hermaphroditum* (Schwarz et al. 2025), *C. becei* (Correa et al. 2025), *C. elegans* (Kim et al. 2022), *C. remanei* (Teterina et al. 2020) and *Oscheius onirici* (Wellcome Sanger Institute). In each, the X chromosomes are composed of a mosaic of ancestral segments (Fig. S1) (Tandonnet et al. 2019; Foster et al. 2020; Gonzalez de la Rosa et al. 2021). Data from the publicly available sources are a mix of Hi-C and restriction enzyme independent Omni-C (see file S1), both of which are able to detect TADs (Lee et al. 2022; Kurbidaeva et al. 2025). Condensin I-DC is expressed and performs dosage compensation in the somatic cells of hermaphrodites and females (Kramer et al. 2016). Therefore, we performed our Hi-C in early stage larvae, before germline cells begin to proliferate (see Materials and Methods).

While analyzing our Hi-C data mapped to the *P. pacificus* genome, we noticed several misjoins and inversions on chromosomes V and X (Fig. S4). There were a total of 41 small, unplaced contigs (median length = 43,467 bp), many of which displayed contacts with the X chromosome. With our HiC data, we were able to fix the misjoins and inversions on chromosomes V and X, and placed pbcontig517 on the left arm of the X chromosome (Fig. S4). We also lifted over the annotations of protein coding genes. We re-mapped our *P. pacificus* Hi-C reads to the updated assembly.

We assessed the presence of loop-anchored TADs by three criteria (Fig. 2a). First, TADs are observed as “squares” of varying intensity and size on a Hi-C matrix. Boundaries separating the associating domains show a dip in the insulation score, which is computed by sliding a diamond shaped window along the diagonal and summing up the contacts within the window for each position (Open2C et al. 2024). At boundaries, insulation scores dip as a result of lowered contact frequency between upstream and downstream loci (e.g. between two TADs). Second, the two boundaries (e.g. *rex* sites) of a loop-anchored TAD interact in 3D space and are observed as a focal enrichment off the diagonal (corner peak/dot). Third, a stripe, which represents interactions between a boundary and other locations within a TAD is sometimes observed due to loop-extrusion activity of the SMC complex being blocked on one site.

In *C. elegans*, condensin I-DC forms loop-anchored TADs that are observed specifically on the hermaphrodite X chromosome (Figs. 2b and S5). In *C. becei* and *C. remanei*, all DCC subunits have orthologs, and TAD structures are observed specifically on the X chromosomes (Fig. 2b). The less defined TAD features of *C. remanei* are expected because the Hi-C data were obtained from mixed sex (1:1) and mixed stage worms, diluting the female and soma-specific features of dosage compensation with male and germ cells. While the *C. becei* data are also mixed sex (1:1), data were collected in L4 stage larvae (Solomon Sloat, personal communication), which have fewer germline cells and embryos than mixed stage populations. TAD features are more prominent when the observed Hi-C contact matrix is normalized to the matrix expected by genomic distance. In the *C. remanei* data, observed/expected plots reveal the loop anchors (Fig. S6).

Strikingly, *P. pacificus* also displayed loop-anchored TADs on the hermaphrodite X chromosome (Figs. 2b, S5, and S6). While *P. pacificus* has fewer TADs compared to *C. elegans* (Fig. S7), its X chromosome is less assembled, which may be affecting their detection. Loop-anchored TADs were absent on the autosomes of all species (Figs. 2b and S6). Therefore, in *C. becei*, *C. elegans*, *C. remanei*, and *P. pacificus*, loop-anchored TADs are specific to the X chromosomes.

### Distance Decay Analysis Supports the Presence of an X-Specific Loop Extruding Factor in *P. pacificus*

**Fig. 3.**
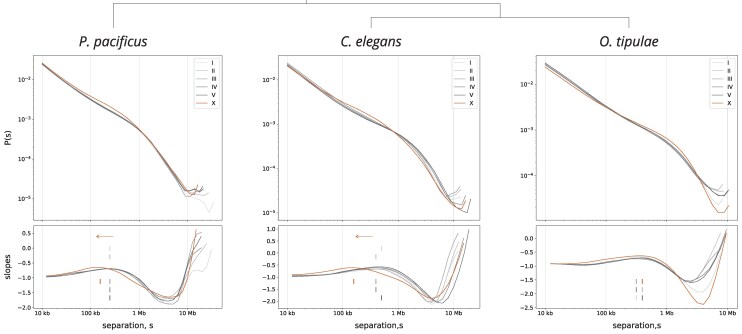
Distance decay curve in *P. pacificus* supports the presence of an X-specific loop extruder similar to the *C. elegans* dosage compensation condensin. Distance decay curves in each species show the contact probability, *P*(s), as a function of separation, s, for each chromosome at 5 kb resolution (top panel). Mean loop sizes for each chromosome were computed by taking the local maxima of the slope (bottom panel, tick marks). Similar to *C. elegans*, the *P. pacificus* hermaphrodite X chromosomes display a local maxima shifted to the left (arrow) when compared with the autosomes, indicative of an X chromosome–specific loop extruding factor, and an increase in the proportion of smaller loops on the X. *O. tipulae* does not show a difference in mean loop size between the X chromosome and autosomes, consistent with a lack of X-specific loop extruding activity.

We also assessed the presence of an X -specific SMC complex independent of calling TADs. The loop extrusion activity of condensin I-DC on the X chromosomes is detected by analyzing the decay of DNA contacts as a measure of increased distance, P(s) (Kim et al. 2022; Morao et al. 2022) (Fig. 3, top panel). Condensin I-DC-mediated DNA loops produce a characteristic shoulder on the distance decay of 3D contacts from the X chromosome compared with the autosomes (Crane et al. 2015; Gassler et al. 2017; Kim et al. 2022; Morao et al. 2022). We performed the same analysis on our *P. pacificus* Hi-C data and found that the X chromosome shows the characteristic shoulder not found on the autosomes (Figs. 3 and S8).

Modeling of loop extrusion factors (LEFs) suggests that the local maxima of the log derivative of the P(s) corresponds to the mean loop size, which is decreased by increasing the number of LEFs (Goloborodko et al. 2016; Gassler et al. 2017; Polovnikov et al. 2023). Indeed, the mean loop size of the *P. pacificus* X chromosome shows the same shift to the left observed in *C. elegans* (Figs. 3 and S8). To test if the mean loop size was statistically different between the X chromosome and autosomes, we performed a permutation test, which supported the presence of an X-specific loop extruder in *P. pacificus* (Fig. S9). As our test statistic, we collapsed the autosomes, and measured the difference in mean loop size between the autosomes and X chromosomes. In *P. pacificus* and *C. elegans*, the absolute value of the permuted test statistic was greater than the absolute value of the observed in 0.89% and 0.37% of permutations, respectively. In addition, a phylogenetic analysis of cohesin, condensin, and SMC-5/6 subunits found no other SMC duplications (Fig. S10). Taken with the presence of loop-anchored TADs (Fig. 2b) and a diverged SMC-4 variant (Fig. 1d), our data suggest that *P. pacificus* contains an X chromosome–specific condensin complex.

### Hi-C Analysis Indicates No X-Specific Loop-Anchored TADs in *O. tipulae*, *O. onirici*, and *S. hermaphroditum*

Unlike *P. pacificus*, *O. tipulae* does not contain paralogs of SMC-4 (Fig. 1c). Importantly, our Hi-C data in *O. tipulae* did not display loop-anchored TADs on the X chromosome (Figs. 2b, S5, and S6). Consistent with the lack of an X chromosome–specific loop extruder in *O. tipulae*, there was no difference between mean loop size on the X and autosomes (Figs. 3 and S8). We also analyzed the publicly available Hi-C dataset for *O. onirici* (Wellcome Sanger Institute) and *S. hermaphroditum* (Schwarz et al. 2025), obtained from mixed stage, hermaphrodite animals. While the mixed stage *and* mixed sex *C. remanei* data show loop-anchored TADs, we observed no TADs in the less diluted (mixed developmental stage, but not sex) *O. onirici* and *S. hermaphroditum* matrices (Figs. 2b and S6). Together with our phylogenetic analysis, these Hi-C results place *Oscheius* and *Steinernema* in the lineages lacking an X-specific loop extruder.

### *O. tipulae* X Chromosomes Are Dosage Compensated

In *O. tipulae*, the absence of an SMC-4 paralog and loop-anchored TADs could either be because this species does not compensate or because it uses an alternate mechanism. Using mRNA-seq data from males and hermaphrodites, we previously established that *P. pacificus* and *C. remanei* X chromosomes are dosage compensated (Albritton et al. 2014). To test if *O. tipulae* dosage compensates, we analyzed publicly available RNA-seq data (Dockendorff et al. 2022) and applied the same criteria established for assessing dosage compensation in *C. elegans* (Albritton et al. 2014; Kramer et al. 2016).

**Fig. 4.**
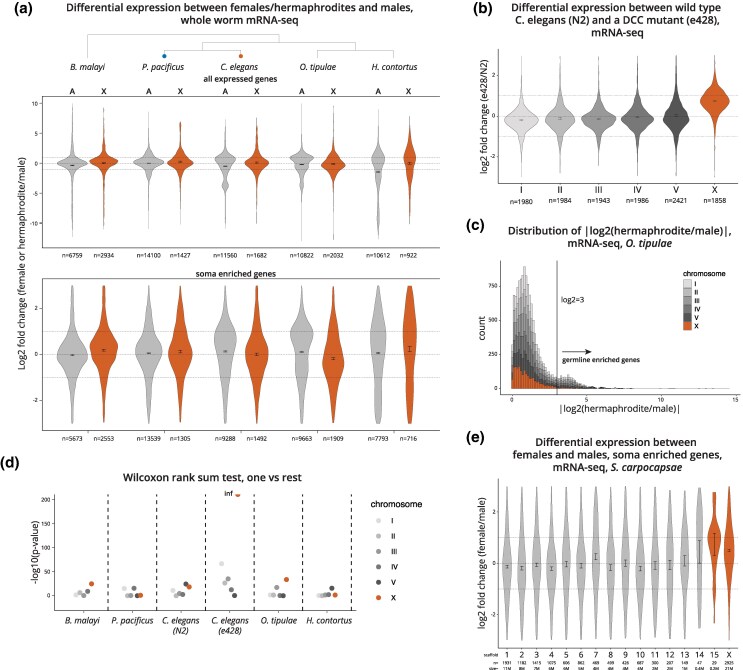
X chromosomes are consistently dosage compensated in multiple nematode lineages. a) Log_2_ fold difference in gene expression between females (or hermaphrodites) and males is plotted as a violin plot for expressed genes (mean TPM of replicates > 1 in both sexes) on autosomes, A, and X chromosomes, X (top panel), and in soma enriched genes (bottom panel). Bars represent confidence intervals. Blue and orange circles are placed on the tree where SMC-4 duplications occurred in *Pristionchus* and *Caenorhabditis*, respectively. b) Log_2_ fold difference between wild-type *C. elegans* (N2) and a dosage compensation mutant, e428, showing the expected level of expression on the X chromosomes in the absence of dosage compensation by the DCC. c) Distribution of sex-biased gene expression from adults plotted as |log_2_(hermaphrodite/male)| in *O. tipulae*. Similar to *C. elegans*, a bimodal distribution of sex-biased gene expression in *O. tipulae* results from germline-enriched genes with higher sex-biased gene expression. d) −Log_10_(*P*-value) from two-sided Wilcoxon rank sum tests comparing the mean log_2_ fold difference of a chromosome to the mean of the rest (e.g. mean of X and mean of I to V). *P*-values in all but the e428 mutant cluster, suggesting no statistical difference between each chromosome in each wild-type species. Inf, infinite. e) Log_2_ fold difference between females and males in *S. carpocapsae* in soma enriched genes separated by scaffold/chromosome does not support chromosome-wide dosage compensation of the X in this species. Scaffold 15 is colored orange because of its predicted location on the X chromosome (Serra et al. 2019). *n*, number of genes in a), b), and e). Size of each chromosome is shown in megabases (M).

Dosage compensation mechanisms control the balance between the X and autosomes, and equalize X chromosomal transcript levels between sexes (Deng et al. 2011; Ercan 2015). If there is dosage compensation, the average ratio of mRNA expression between sexes should be similar between the X chromosomes and autosomes. We analyzed mRNA-seq data in *B. malayi*, *O. tipulae*, and *Haemonchus contortus* adults and reanalyzed data from *P. pacificus* young adults and *C. elegans* adults (Laing et al. 2013; Albritton et al. 2014; Chung et al. 2018; Doyle et al. 2020; Dockendorff et al. 2022). In each case, the average ratio of gene expression between sexes on the X and autosomes was similar (Fig. 4a, top panel). In contrast, the same approach detects the difference between X and autosomal expression in the larvae of a dosage compensation mutant *C. elegans* strain (*dpy-21(y428)*) (Fig. 4b) (Kramer et al. 2015).

In *C. elegans*, dosage compensation occurs in the somatic cells, but not in the germ cells, which can make up to two thirds of all cells in adults (Strome et al. 2014; Kramer et al. 2016). In addition, genes expressed in the germline are underrepresented on the X chromosomes due to nonsex-specific silencing during meiosis (Reinke et al. 2004; Wang et al. 2009). To evaluate dosage compensation in somatic cells, we used our previous strategy of enriching for soma expressed genes based on a cutoff selected by the bimodal distribution of differential expression between sexes (Albritton et al. 2014).

In *C. elegans*, genes expressed specifically in the germ cells often show >8-fold difference between hermaphrodites (or females) and males (Albritton et al. 2014). Therefore, using a cutoff of log_2_ fold change = 3 removes most germline-enriched genes (Albritton et al. 2014). Similar to *C. elegans*, the absolute log_2_ fold change between sexes shows a bimodal distribution in *O. tipulae* (Fig. 4c). The level of germ-cell driven sex-biased gene expression observed in the mRNA-seq data from whole worms depends on the number of germ cells in the worms (e.g. more in the adults than in L3 larvae) (Kramer et al. 2016). We observed varying levels of sex-biased gene expression in *B. malayi* and *H. contortus*, which may be due to differences in the number of germ cells present in the sample or varying levels of differential expression in the germ cells (Fig. S11). Regardless, using the same cutoff across species is a conservative approach to enrich for soma-expressed genes, resulting in a lower difference between sexes (Fig. 4a, bottom panel) and less variation between autosomes (Fig. S12).

Each autosome harbors thousands of different genes with varied sex-biased gene expression (Figs. S11 and S12). We used this variability to statistically test for the presence of X chromosome dosage compensation in *O. tipulae*. We employed a “one versus rest” approach, comparing the mean fold difference between sexes on each chromosome to the rest (e.g. I vs. II, III, IV, V, and X). Similar to *C. elegans* and *P. pacificus*, in *O. tipulae* we found the difference in sex-biased gene expression between the X and autosomes to be no more than the difference between an autosome and the rest of the chromosomes (Fig. 4d). In contrast, in the *C. elegans* dosage compensation mutant (*dpy-21(e428)*), the difference between the X and autosomes is readily apparent. Therefore, X chromosomes are dosage compensated in *O. tipulae*. Together with *B. malayi* and *H. contortus*, these results suggest that dosage compensation is older than condensin I-DC in nematodes.

### *Steinernema carpocapsae* X Chromosomes Are Not Completely Dosage Compensated

Similar to *O. tipulae*, *S. hermaphroditum* does not have an SMC-4 duplicate or X-specific loop-anchored TADs. To evaluate if there is dosage compensation in the *Steinernema* lineage, we analyzed publicly available mRNA-seq data in female and male young adults in *S. carpocapsae* (Rodriguez et al. 2020). Although the *S. carpocapsae* genome assembly is not chromosome scale, the X chromosome is assembled, and the remaining four autosomes are split among 14 scaffolds and contigs (Serra et al. 2019). To our surprise, we found that expression in females is higher than males on the X chromosome (log2 fold change > 0), which could not be explained by the variation among the autosomes (Figs. 4e and S13). It is possible that *S. carpocapsae* X chromosomes are not fully dosage compensated like the other nematode species we analyzed (Fig. 4a).

*Steinernema* X chromosomes underwent two recent fusions, once to Nigon element B, and once to D (Gonzalez de la Rosa et al. 2021). Furthermore, Nigon elements B and D make up a higher proportion of the X chromosome than Nigon element X (Fig. S1). Therefore, we considered the possibility that dosage compensation is complete on Nigon element X, but incomplete or absent on elements B and D. However, we observed that the expression of genes on all three elements were higher in females than males (Fig. S14), excluding the possibility of complete dosage compensation specifically on the ancestral X element.

### H4K20me1 Is Enriched on the X Chromosomes in Both *O. tipulae* and *P. pacificus*

**Fig. 5.**
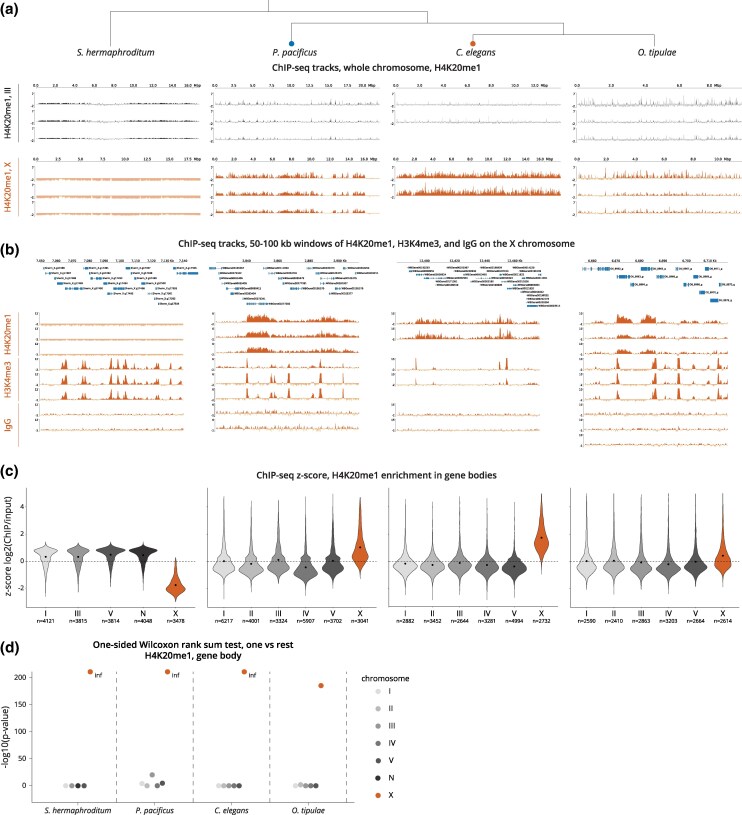
*P. pacificus* and *O. tipulae* X chromosomes are enriched for H4K20me1. a) H4K20me1 ChIP-seq enrichment tracks (ChIP minus input) are shown for the entire X chromosome and a representative autosome, III in *S. hermaphroditum*, *P. pacificus*, *C. elegans*, and *O. tipulae* hermaphrodite larvae. Two to three biological replicates are shown per species. Negative values on each track are due to normalization to input data and are faded. b) Representative 50 to 100 kb windows are shown to highlight the distribution of H4K20me1, H3K4me3, and IgG ChIP-seq tracks (ChIP minus input) relative to gene locations. Genes are annotated above the tracks. c) Replicates were merged and H4K20me1 ChIP enrichment across gene bodies (*z*-scored log_2_(ChIP/input)) was plotted as a violin plot for each chromosome. H4K20me1 levels are higher on the X chromosomal gene bodies of *P. pacificus*, *C. elegans*, and *O. tipulae* when compared to autosomes. In contrast, H4K20me1 is depleted on the X chromosomal gene bodies of *S. hermaphroditum*. Black dots represent the mean. *n*, number of genes. d) −Log_10_(*P*-value) from one-sided Wilcoxon rank sum tests comparing the mean log2(ChIP/input) of a chromosome to the mean of the rest (e.g. mean of X and mean of I to V). Statistics were run on the merged data.

In *C. elegans*, X chromosome repression is mediated by condensin I-DC and DPY-21 (Vielle et al. 2012; Wells et al. 2012; Brejc et al. 2017). The demethylation of H4K20me2 by DPY-21 results in the enrichment of H4K20me1 on the X chromosomes when compared to autosomes (Vielle et al. 2012; Wells et al. 2012; Brejc et al. 2017). The repression of the X chromosomes also results in the depletion of histone modifications associated with active transcription (Street et al. 2019). We wondered if *O. tipulae* and *P. pacificus* show X-specific differences in histone marks associated with dosage compensation in *C. elegans* (Vielle et al. 2012; Wells et al. 2012; Street et al. 2019). We performed ChIP-seq analysis of H4K20me1, H3K4me3, and as a negative control, IgG in early stage larvae prior to germline proliferation. We found that similar to *C. elegans*, H4K20me1 is elevated across the entire X chromosome relative to autosomes in both species (Figs. 5a and S15).

H4K20me1 and H3K4me3 ChIP-seq data shown across a representative region highlight the gene body and promoter enrichment of H4K20me1 and H3K4me3, respectively (Fig. 5b). This suggests that the functions of these modifications are conserved. Analyzing relative enrichment of H4K20me1 on each chromosome indicated that H4K20me1 is enriched on the X chromosomal gene bodies of each species (Figs. 5c and S16). We validated that the low enrichment of H4K20me1 on the *O. tipulae* X chromosomes is statistically significant by comparing average gene body ChIP-seq *z*-scores in each chromosome to the rest (Fig. 5d). Given that H4K20me1 enrichment in *C. elegans* is not limited to gene bodies, we also analyzed relative enrichment of H4K20me1 chromosome-wide and in intergenic regions. In both, we observed a similar enrichment specific to the X chromosome (Figs. S17 and S18). The enrichment of H4K20me1 in *O. tipulae* is in contrast to the Hi-C data, where loop-anchored TADs are present in *P. pacificus*, but not *O. tipulae*. It is possible that *O. tipulae* has a mechanism of X chromosome repression that employs H4K20 monomethylation without condensin.

### H4K20me1 Is Depleted on the Hermaphrodite X Chromosomes of *S. hermaphroditum*

In *S. hermaphroditum*, TADs were absent from the hermaphrodite X chromosome, and in *S. carpocapsae*, we did not find evidence of chromosome-wide dosage compensation. In the possible absence of dosage compensation, we predicted that the *S. hermaphroditum* X chromosome would not be enriched for H4K20me1. ChIP-seq analyses of H4K20me1, H3K4me3, and IgG in *S. hermaphroditum* early stage hermaphrodite larvae showed that the X chromosomes were “depleted” for H4K20me1, and surprisingly, enriched for H3K4me3 (Figs. 5, S15 to S18). This pattern of ChIP-seq enrichment was not a result of the unequal distribution of genes among the chromosomes (Fig. S19). Lack of H4K20me1 enrichment on the X chromosomes is consistent with both the absence of TADs *and* the absence of chromosome-wide dosage compensation in *Steinernema*.

## Discussion

**Fig. 6.**
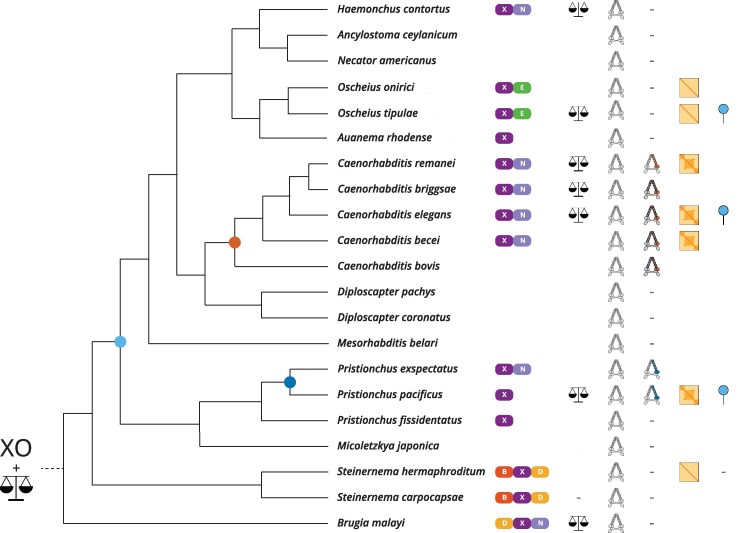
Model for the evolution of dosage compensation in nematodes. Nematodes display significant changes to X chromosome content resulting from autosome to sex chromosome fusions (column 1). Dosage compensation is as old as the common ancestor of Spirurina, Tylenchina, and Rhabditina, which is supported by its presence in *Brugia*, *Pristionchus*, *Caenorhabditis*, *Haemonchus*, and *Oscheius* (column 2). Here, we postulate that it is likely as old as the XO sex determination in nematodes. The canonical SMC-4 is found in all nematodes sampled (column 3). DPY-27, the condensin I-DC specific subunit, is an SMC-4 paralog found only in *Caenorhabditis* (column 4), and the duplication event occurred in the lineage leading to *Caenorhabditis* (orange circle). A second, independent SMC-4 duplication occurred in *Pristionchus* (column 4, dark blue circle). X chromosome–specific loop-anchored TADs are a signature of condensin-mediated dosage compensation in *C. elegans*, and are also observed in *P. pacificus*, *C. becei*, and *C. remanei*, but not in *S. hermaphroditum*, *O. tipulae* and *O. onirici* (column 5). Here, we postulate that X-specific condensins in nematodes evolved in parallel in *Caenorhabditis* and *Pristionchus*. The repressive histone mark, H4K20me1, is enriched on the hermaphrodite X chromosomes without SMC-4 duplication or TADs in *O. tipulae*, suggesting it precedes condensins as a mechanism common to Rhabditina (column 6, light blue circle). -, not found; blank space, not checked; 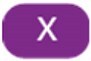, Nigon element; 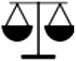, chromosome-wide dosage compensation; 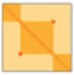, X-specific TADS; 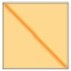 no TADs on X chromosome; 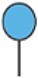, X-specific enrichment of H4K20me1.

In this study, we found dosage compensation mechanisms continue to change within a clade of shared X chromosome ancestry. We also found that the *C. elegans* condensin I-DC evolved recently, in the lineage leading to *Caenorhabditis* and in the presence of an existing mechanism of dosage compensation (Fig. 6). This mechanism seems to have its basis in the conserved histone demethylase, DPY-21 and may have been present in the common ancestor of Rhabditina. Intriguingly, a condensin-based mechanism to regulate X chromosome structure evolved independently in *Pristionchus*, which is indicative of common selective pressures acting on dosage compensation, and a constraint on the evolution of dosage compensation in nematodes.

### XO Sex Determination and Dosage Compensation Are Ancient in Nematodes

The degree of Y chromosome degeneration in XY sex determination systems can be a powerful predictor of dosage compensation. For example, in the fish genus *Poecilia*, dosage compensation is found in species with heteromorphic, but not homomorphic sex chromosomes (Metzger et al. 2021). In nematodes, the complete loss of the Y chromosome is ancestral, although the Y chromosome can reevolve by fusion of the X chromosome with an autosome as observed in *B. malayi* (Foster et al. 2020). Analyses of differential gene expression between sexes in *B. malayi*, *O. tipulae*, and *H. contortus* suggest that each of their X chromosomes are dosage compensated (Fig. 4), as expected in an ancient XO sex determination system.

Surprisingly, the *S. carpocapsae* expression data do not support chromosome-wide dosage compensation of its X chromosomes (Fig. 4e). We do note that the observed difference in expression between females and males is <2-fold (log_2_ fold difference = 1.4). It is possible that X chromosomal dosage-sensitive genes are compensated on a gene-by-gene basis or that a general buffering mechanism partially compensates for X chromosome dosage difference between sexes (Zhang et al. 2010; Malone et al. 2012). In addition, despite our attempt to remove germline expressed genes, it is possible that some germline expression contributed to the less than 2-fold difference. Finally, we are careful to not suggest that dosage compensation is absent in *S. carpocapsae*, but is incomplete.

A parsimonious view of the expression data, along with the age of Y chromosome degeneration in nematodes, suggests that chromosome-wide dosage compensation was present in the common ancestor of Rhabditina, Tylenchina, and Spirurina, but was partially lost in the lineage leading to *Steinernema*. This loss may be explained by two significant autosome to sex chromosome fusions in *Steinernema* (Fig. S1). We observed that sex-biased gene expression on Nigon X in *S. carpocapsae* is not significantly different from the fused Nigon elements B and D (Fig. S14). Therefore, it is possible that the fusions interfered with the existing mechanism on Nigon X. We also consider the possibility that the common ancestor of Rhabditina, Tylenchina, and Spirurina dosage compensated on a gene-by-gene basis, and there were two gains of chromosome-wide dosage compensation, one in Spirurina and one in Rhabditina. In both possibilities, we postulate that dosage compensation of either kind is older than the common ancestor of Rhabditina, Tylenchina, and Spirurina, and concurrent with the XO sex determination system (Fig. 6).

### Condensin-Mediated Dosage Compensation Evolved Recently in the Lineage Leading to *Caenorhabditis*

Our phylogenetic analysis shows that condensin I-DC arose through duplication and divergence of SMC-4 in the lineage leading to *Caenorhabditis* (Fig. 1). In *C. elegans*, the presence of looped anchored TADs is a molecular phenotype of condensin I-DC activity (Kim et al. 2022). While X-specific TADs were found in *C. becei* and *C. remanei*, no such TADs were found in *O. tipulae*, *O. onirici*, and *S. hermaphroditum*, which lack an SMC-4 paralog (Figs. 2b and 6). Together, these results suggest that condensin I-DC is a newly evolved mechanism of dosage compensation.

Changes to sex chromosome composition are common in nematodes and may explain the inception of condensin I-DC in *Caenorhabditis* (Foster et al. 2020; Gonzalez de la Rosa et al. 2021). In *Drosophila*, autosome to sex chromosome fusions are followed by degeneration of the nonfused homolog, leaving the neo X-chromosomal genes in one copy (Bone and Kuroda 1996; Marín et al. 1996; Ellison and Bachtrog 2019). This dosage problem was solved by evolution of DNA sequence motifs that recruit the existing DCC to the neo-X chromosomes. In contrast, different mechanisms of dosage compensation seem to be acting on the neo and ancestral segments of the Z chromosome in the Monarch butterfly, *Danaus plexippus* (Gu et al. 2019). In *D. plexippus*, while the neo-segment of the Z chromosome is upregulated in females (ZW) as in *D. melanogaster*, the ancestral segment of the Z chromosome is downregulated in males (ZZ) as in *C. elegans* (Gu et al. 2019). Therefore, new mechanisms of X chromosome regulation may evolve in the presence of an old one.

Our data support the idea that *C. elegans* condensin I-DC evolved in the presence of an existing mechanism in the nematode clade. It is possible that an X chromosome event was the selective pressure to evolve condensin I-DC, such as the X to N fusion in the lineage leading to *Caenorhabditis* (Fig. S1) (Tandonnet et al. 2019; Foster et al. 2020; Gonzalez de la Rosa et al. 2021). Such an event is not obvious in *Pristionchus* where a second X-specific SMC complex has evolved. Nevertheless, while the X chromosomes are largely syntenic in *C. elegans* and *C. briggsae*, the DNA sequences that recruit the DCC to the X chromosomes functionally diverged between the two species (Yang et al. 2023). Therefore, large chromosomal events are not required for the continual evolution of the dosage compensation machinery.

### Parallel Evolution of Condensin-Mediated Dosage Compensation in *P. pacificus*?

In perhaps the most surprising observation, we identified an independent duplication of SMC-4 in the common ancestor of *P. pacificus* and *P. exspectatus* (Figs. 1 and 6). Hi-C analysis in *P. pacificus* revealed X-specific loop-anchored TADs in hermaphrodite larvae (Fig. 2b). An exploration of the other SMC proteins, namely cohesin, condensin I, condensin II, and SMC-5/6 did not reveal any other duplication events in *Pristionchus* (Fig. S10). A multiple sequence alignment of the conserved ATPase domains implicated one group of SMC-4 paralogs in dosage compensation (Fig. 1d). The independent duplication and divergence of SMC-4 toward a possible dosage compensation function in *Caenorhabditis* and *Pristionchus* suggests a common selective pressure in these two lineages. It will be important to determine what constraints lead to the parallel evolution of condensin-based mechanisms of dosage compensation in nematodes.

SMC genes are already known to produce new genes by duplication. In fact, the entire SMC family of genes arose from a common SMC ancestor through several rounds of duplication and neofunctionalization (Hirano 2002; Cobbe and Heck 2004; Yoshinaga and Inagaki 2021; Van Hooff et al. 2025). Interestingly, an SMC protein for dosage compensation evolved in not one but “two” clades with independent X chromosome ancestry. The first is the nematode condensin I-DC, and the second is SMCHD1 in mammals (Blewitt et al. 2008). Similar to the *C. elegans* DPY-27, SMCHD1 functions in 3D organization and repression of the inactivated X chromosomes in mice and humans (Blewitt et al. 2008; Sakakibara et al. 2018; Gdula et al. 2019; Wang et al. 2019; Bowness et al. 2022). It is possible that the chromosome-wide binding capabilities of SMC complexes are well suited to solve the chromosome-wide transcriptional imbalance that necessitates dosage compensation.

### Does a Mechanism Based on H4K20me1 Predispose Rhabditina to Evolve Condensin-Mediated Dosage Compensation?

ChIP-seq data in *O. tipulae* show that H4K20me1 is enriched on the X chromosome despite the absence of an SMC-4 duplication and X-specific loop-anchored TADS. In *C. elegans*, H4K20me1 is enriched by DPY-21, which is recruited by condensin I-DC to the X chromosomes in mid embryogenesis (Vielle et al. 2012; Wells et al. 2012; Brejc et al. 2017). H4K20me1 enhances X chromosome repression for dosage compensation (Plenefisch et al. 1989; Davis and Meyer 1997; Kramer et al. 2015; Brejc et al. 2017). In *C. elegans*, DPY-21 also functions in the germline without condensin I-DC, where it is required for increasing H4K20me1 on the autosomes, but not the X chromosome (Brejc et al. 2017).

Our results suggest that H4K20me1 enrichment preceded the SMC-4 duplications in Rhabditina. The alternative interpretation that SMC-mediated dosage compensation preceded H4K20me1 requires that the SMC-4 duplicate be lost in *O. tipulae* without the complete loss of H4K20me1. This is less likely because based on the phylogeny of the species analyzed (Fig. 6), the SMC-4 duplicate would need to be lost four times and gained once: lost in the common ancestor of the *Diplogastrids* followed by a gain in the common ancestor of *P. pacificus* and *P. exspectatus*, lost in the branches leading to *M. belari* and *Diploscapter*, and lost in the common ancestor of *Auanema*, *Oscheius*, *Necator*, *Ancylostoma*, and *Haemonchus*.

H4K20me1 is a highly conserved histone modification that is enriched on mitotic chromosomes when canonical condensins are active (Beck et al. 2012). We speculate that a mechanistic link between H4K20me1 and condensin I contributed to the process of condensin I-DC evolution. Canonical condensin I remains cytoplasmic until the nuclear envelope breaks down during cell division (Ono et al. 2004; Shintomi and Hirano 2011). Perhaps the nematode condensin I bound and increased H4K20me1 on the X chromosomes during interphase, while also functioning to compact all chromosomes during mitosis. Condensin I's dual role in dosage compensation and mitosis may have presented two possibly conflicting constraints. Duplication of the SMC-4 subunit followed by divergence would alleviate one of its constraints, allowing for the separate evolution of two condensin I variants.

Interestingly, an additional SMC may have evolved in *Caenorhabditis* to further mediate the potential interaction between condensin I and I-DC. Mass spectrometry analysis of proteins that interact with DPY-27 found a small SMC-like protein 1 (SMCL-1) in *C. elegans* (Chao et al. 2017). SMCL-1 lacks the SMC hinge and coils, contains a nonfunctional ATPase domain, and negatively regulates condensin I and I-DC (Chao et al. 2017). Future studies should address if parallel evolution of the SMC-4 paralog in *P. pacificus* also necessitated the emergence of a negative regulator of condensin I.

### Concluding Remarks

While the field has been able to assess whether X chromosomes are dosage compensated or not by using differential expression analysis between sexes, the mechanisms of dosage compensation have been traditionally addressed in model organisms representing just a few clades. Recent efforts have been made to begin characterizing the mechanisms of dosage compensation in less represented groups like butterflies, moths, mosquitoes, fish, birds, and lizards (Marin et al. 2017; Gu et al. 2019; Krzywinska et al. 2021; Rosin et al. 2022; Kalita et al. 2023; Tenorio et al. 2024; Fallahshahroudi et al. 2025). By combining a phylogenetic analysis of the *C. elegans* DCC with a comparative analysis of its genomic, transcriptomic, and epigenomic signatures, our work found that a new dosage compensation mechanism evolved in the presence of an existing one in the *Caenorhabditis* lineage.

The existing dosage compensation mechanism in the common ancestor of *O. tipulae*, *C. elegans*, and *P. pacificus* may be linked to H4K20me1, and may have constrained evolution toward an X-specific condensin in *Caenorhabditis* and *Pristionchus*. Furthermore, the observation that SMC proteins repeatedly evolved for dosage compensation in nematodes *and* mammals argues for a fundamental link between the chromosome-wide activity of SMC complexes and the need to regulate transcription across the entire X chromosome. A more exhaustive accounting of dosage compensation phenotypes in multiple species spanning “all” five clades would help delineate the history of sex chromosome dosage compensation in nematodes and potentially uncover new strategies for chromosome-wide gene regulation.

## Materials and Methods

### Phylogenetic Analysis

Datasets used can be found in file S1. To assess annotation quality, BUSCO scores were called in protein mode against the nematoda lineage. We used version 3.0.2 (OBD9 dataset) for the diplogastrids and version 5.3.0 (ODB10 dataset) for the rest (Waterhouse et al. 2018; Rödelsperger 2021; Manni et al. 2021a, 2021b). We called orthologs using OrthoFinder (version 2.5.4) by first extracting the longest transcript per gene with the provided primary_transcripts.py script and then running OrthoFinder with the default parameters (Emms and Kelly 2015; Emms and Kelly 2019). We removed orthologs that were less than half the size of SMC-4 in *C. elegans*. These sequences represent either partial duplications or duplications followed by deletions and are unlikely to have the function of SMC-4 preserved (see Table 2). Furthermore, the resulting gaps in the alignment would affect downstream analysis. We aligned orthologs using MAFFT (version 7.475) with the following parameters: --local pair --maxiterate 1000 (Katoh and Standley 2013). We generated a maximum-likelihood gene tree using IQ-Tree (version 2.2.0) on the aligned sequences with the following parameters: --seqtype AA -m MFP -B 1000 (Nguyen et al. 2015; Kalyaanamoorthy et al. 2017; Hoang et al. 2018). The trees were rooted and edited in FigTree (version 1.4.4) and TreeViewer (Bianchini and Sánchez-Baracaldo 2024).

### Nigon Painting

Datasets used can be found in file S1. BUSCO (version 5.3.0) was run on the assembly against the nematoda lineage in the ODB10 dataset (Waterhouse et al. 2018; Manni et al. 2021a, 2021b). Completementness was assessed, and the full_table.tsv file was used to paint chromosomes online at https://pgonzale60.shinyapps.io/vis_alg/ using the default parameters and by following the instructions in the GitHub repository https://github.com/pgonzale60/vis_ALG (Gonzalez de la Rosa et al. 2021).

### Strains and Growth Conditions

We used the following strains for *O. tipulae*, *P. pacificus*, and *S. hermaphroditum*, respectively: CEW1, PS312, and PS9179. *O. tipulae* and *P. pacificus* were maintained on 6 cm nematode growth medium (NGM) plates (final concentrations of 3 g/L NaCl, 2.5 g/L peptone, 20 g/L agar, autoclave, and then add 1 mM calcium chloride, 5 mg/L cholesterol, 1 mM magnesium sulfate, and 25 mM potassium phosphate buffer) seeded with a streptomycin resistant strain of *Escherichia coli*, OP-50. *S. hermaphroditum* was maintained on 10 cm NGM plates seeded with a kanamycin and streptomycin resistant strain of *Xenorhabdus griffiniae*, HGB2587. Plates were cultured at 22, 20, and 25 °C, respectively.

### Hi-C

*O. tipulae* and *P. pacificus* were propagated for collection on 10 cm NGM plates supplemented with agarose (10 g/L agar, 7 g/L agarose in place of 20 g/L agar) to prevent burrowing and seeded with OP-50. Plates were fed as needed with a concentrated strain of *E. coli*, HB101. For *O. tipulae*, mixed stage plates were washed with sterile M9 and a cell spreader to unstick embryos from the plate. Adults and larvae were killed by nutating in a bleach solution (0.5 M NaOH, 1% sodium hypochlorite) for 2 min, washed with M9, and arrested at L1 by nutating in M9 for 24 h. The worms were filtered through a 15 µm nylon mesh to separate arrested L1 larvae from carcasses and unhatched embryos, and then plated. The worms were cultured for 24 h, fixed by nutating in 2% formaldehyde (in M9) for 30 min, quenched by adding glycine to 125 mM for 5 min, washed with M9, flash frozen in liquid nitrogen, and stored at −80 °C. The extent of germline proliferation after 24 h was determined by differential interference contrast (DIC) microscopy.

For *P. pacificus*, mixed stage plates were filtered through a 20 µm nylon mesh to produce a predominantly J2 to J3 flow through. The flow through was fixed by nutating in 2% formaldehyde (in M9) for 30 min, quenched by adding glycine to 125 mM for 5 min, washed with M9, flash frozen in liquid nitrogen, and stored at −80 °C.

For Hi-C, around 50 µL of pelleted larvae was removed from −80 °C and resuspended in 20 µL phosphate buffered saline (PBS) with 1 mM phenylmethylsulfonyl fluoride (PMSF) added fresh. A mortar and pestle (BioSpec Products, catalog # 206) was placed on dry ice and filled with liquid nitrogen. Drops of larvae were pipetted into the mortar, and pulverized into a fine powder while submerged in liquid nitrogen. The powdered larvae were crosslinked for a second time as described in the Arima Hi-C + High Coverage Kit (catalog # A101030). Hi-C (restriction enzymes: DpnII, HinfI, DdeI, and MseI) was then performed as per Arima's instructions. Library preparation was performed with the KAPA Hyper Prep Kit (KK8502) as per Arima's instructions. Paired-end (100 bp) sequencing was performed at the Genomics Core at the Center for Genomics and Systems Biology, New York University using the Illumina NovaSeq 6000 platform.

### Rescaffolding and Liftover

Hi-C fastq files were mapped to the *P. pacificus* genome (PRJNA12644), and biological replicates were merged using the Arima Genomics mapping pipeline (https://github.com/ArimaGenomics/mapping_pipeline). The output bam file was used to re-scaffold the *P. pacificus* genome with YaHS (version 1.1) (Zhou et al. 2023). The following parameters were used: -r 1000, 2000, 5000, 10000, 20000, 50000, 100000. The draft genome was curated with Juicebox Assembly Tools (JBAT) in Juicebox (see Data Availability) (Durand et al. 2016). Liftoff (version 1.6.3) was used with the default parameters to lift over the protein coding genes to the updated assembly (Shumate and Salzberg 2021).

### Hi-C Data Processing and Analysis

Hic files were generated using Juicer (version 1.5.7) (Rao et al. 2014; Durand et al. 2016). Biological replicates were merged using Juicer's mega.sh script. All output hic files (minimum mapping quality of 30) were converted to cool files using the hicConvertFormat tool in HiCExplorer (version 3.6) with the following parameters: --inputFormat hic --outputFormat cool. Count data were loaded from the cool file using the following parameters: --inputFormat cool --outputFormat cool --load_raw_values. This raw cool file was then balanced using Cooler (version 0.8.11) with the following parameters: --max-iters 500 --mad-max 5 --ignore-diags 2. The balanced cool file was used with Cooltools (version 0.4.0) to generate the Hi-C matrixes, calculate observed/expected and insulation scores, and compute the P(s) and its derivative (see Data Availability) (Open2C et al. 2024). All plots were made with Matplotlib (version 3.4.3) (Hunter 2007).

### RNA-Seq Data Processing and Analysis

We used the nf-core rna-seq pipeline (version 3.12.0) to generate counts and TPM tsv files. The workflow of the pipeline under our parameters was as follows: catenate technical replicates, infer strandedness with Salmon (version 1.10.1), assess the sequencing quality with FastQC (version 0.11.9), remove rRNA reads with sortMeRNA (version 4.3.4), align with STAR (version 2.7.10a), quantify with Salmon (version 1.10.1), and summarize counts and TPM quantification at the gene level with tximport (version 1.16.0). We used the follow flags: -profile singularity, --remove_ribo_rna, --save_non_ribo_reads, --save_reference, --skip_umi_extract, --skip_trimming, --skip_bbsplit_reads, --skip_biotype_qc, --skip_stringie, --skip_deseq2_qc. We ran DESeq2 (version 1.42.0) on the gene counts tsv file in R, and used ggplot2 (version 3.5.0) for all plots (Love et al. 2014; Wickham 2016). We used the gene TPM tsv files to filter out unexpressed genes (mean TPM of replicates >1 in both sexes).

### ChIP-Seq

*O. tipulae* and *P. pacificus* were collected like they were for Hi-C. *S. hermaphroditum* was collected as follows. Thirty infective juveniles (F_0_) were split among 3 to 10 cm NGM plates (10 g/L agar, 7 g/L agarose in place of 20 g/L agar) seeded with HGB2587 and fed as necessary with a concentrated form of the same strain until the second generation (F_1_) reached adulthood. The adults were moved with sterile water onto 9 to 10 cm plates in anticipation of overcrowding in the third generation (F_2_). After a couple of days, when the plates were concentrated with third generation larvae, the worms were filtered through a 30 µm filter to produce a larval population without extensive germline proliferation. They were then fixed by nutating in 2% formaldehyde (in PBS) for 30 min, quenched by adding glycine to 125 mM for 5 min, washed with PBS, flash frozen in liquid nitrogen, and stored at −80 °C. The extent of germline proliferation was determined by DIC microscopy.

Around 100 to 150 µL of pelleted larvae was removed from −80 °C and washed in FA Buffer (final concentrations of 50 mM HEPES/KOH pH 7.5, 1 mM EDTA, 1% Triton X-100, 0.1% sodium deoxycholate, 150 mM NaCl, filter sterilized) with 0.1% sarkosyl, 1 mM PMSF, and 1X protease inhibitors (protease inhibitor cocktail set I—Calbiochem, catalog # 539131) added fresh. Larvae were then dounced 30 times in a glass dounce tissue grinder (VWR, catalog # 22877-280) with pestle type B. Sonication was performed in a Bioruptor ® Pico (catalog # B01060010) to obtain fragments between 200 and 800 bp in length (30 s on, 30 s off, 10 to 15 cycles). Protein concentration was determined using the Bradford assay (Bio-Rad, catalog # 500-0006). For chromatin immunoprecipitation, 1 to 2 mg of extract and 3 to 5 µg of antibody were used (see file S1), and allowed to rotate overnight at 4 °C. nProtein A Sepharose beads (Cytiva, catalog # 17528001) were added and incubation was allowed to continue for 2 h. Beads were washed with FA buffer, FA buffer—1 M NaCl, FA buffer—500 mM NaCl, TEL buffer (final concentrations of 0.25 M LiCl, 1% NP-40, 1% sodium deoxycholate, 1 mM EDTA, 10 mM Tris-HCl, pH 8.0, filter sterilized), and Tris-EDTA, respectively. Immunocomplexes were eluted twice with ChIP elution buffer (final concentrations of 1% sodium dodecyl sulfate, 250 mM NaCl, 10 mM Tris, pH 8.0, 1 mM EDTA) at 65 °C for 15 min at 1,400 rpm. The eluate was then treated with 2 µL of 10 mg/mL proteinase K for 1 h at 50 °C and reverse crosslinked overnight in a 65 °C water bath. DNA was purified the next morning with QIAGEN MinElute PCR Purification Kit (catalog # 28006), and fragment size was checked by agarose gel electrophoresis or TapeStation. The remaining DNA was stored at −20 °C.

Library preparation was performed as follows. To perform end repair, we used half the ChIP DNA and 10 to 30 ng of input DNA, T4 DNA Ligase Reaction Buffer (NEB, catalog # B0202S), 0.4 mM dNTPs, 20 U PNK (Fisher, catalog # FEREK0032), 0.7 U Large Klenow Fragment (NEB, catalog # M0210S), and 6 U T4 DNA Polymerase (Fisher, catalog # FEREP0062) (total volume 33 µL). The reaction was incubated at 20 °C for 30 min and was cleaned with QIAGEN MinElute PCR Purification Kit (eluted in 21 µL). To perform A-tailing, NEBuffer 2 (catalog # B7002S), 0.2 mM dATP, and 7.5 U of Klenow Fragment (3′ → 5′ exo-) (NEB, catalog # M0212L) were added to the cleaned DNA (total volume of 25 µL), and incubated for an hour in a 37 °C water bath. The reaction was cleaned with QIAGEN MinElute PCR Purification Kit (eluted in 17 µL). To perform adapter ligation, Quick Ligation Buffer, Illumina TruSeq Adapters (see file S1), and 2 µL of Quick Ligase (NEB, catalog # M2200L) were added to cleaned DNA (total volume of 40 µL), and incubated for 20 min at 23 °C. The reaction was size selected and cleaned with Ampure XP beads (Beckman Coulter, catalog # A63881). The eluate was amplified by PCR with Phusion HF Reaction Buffer, 0.5 µM TruSeq forward and reverse primers (see file S1), 0.2 mM dNTP, and 1 U Phusion High-Fidelity DNA Polymerase (Fisher, catalog # F530L) for a total reaction volume of 50 µL under the following parameters: 98 °C for 1 min, 98 °C for 30 s, 60 °C for 30 s, 72 °C for 30 s, repeat steps 2 to 4 for 14 times, 72 °C for 5 min, 8 °C forever. 10 µL of 3 M sodium acetate (pH 5.2) was added to reaction, and the reaction was cleaned with QIAGEN MinElute PCR Purification Kit (eluted in 11 µL). The eluate was gel purified (1.5% agarose gel) to obtain fragments between 250 to 600 bp using the QIAGEN MinElute Gel Extraction Kit (catalog # 28606). Library concentration was determined with the KAPA Library Quantification Kit (catalog # KK4824). Single-end (75 bp) sequencing was performed at the Genomics Core at the Center for Genomics and Systems Biology, New York University using the Illumina NextSeq 500 platform.

### ChIP-Seq Data Processing and Analysis

Adapters were trimmed when necessary with trimmomatic (version 0.39) (Bolger et al. 2014). Reads were aligned with bowtie2 (version 2.4.2) using the default parameters (Langmead and Salzberg 2012). Sam files were converted to bam files, sorted and indexed using samtools (version 1.11) (Danecek et al. 2021). Bam files were used to generate bigwig files with the bamCoverage tool in deepTools (version 3.5.0) with the following parameters: --binsize 10 --minMappingQuality 20 --extendReads 200 --ignoreDuplicates --normalizeUsing CPM --exactScaling --ignoreForNormalization chrM (Ramírez et al. 2016). Enrichment was normalized to input with the bigwigCompare tool in deepTools. Bigwig files were merged with the bigWigMerge tool in deepTools. Genome browser tracks were made with the hicPlotTADs tool in HiCExplorer (version 3.6) (Ramírez et al. 2018; Wolff et al. 2018, 2020). Enrichment at transcription start sites (TSSs), gene bodies (start to end coordinate), and intergenic regions were computed with the multiBigwigSummary tool in deepTools using the BED-file command. TSS sites were defined as 250 bp upstream and downstream of the first base pair. Intergenic regions were annotated using the complement tool in bedtools (version 2.29.2) (Quinlan and Hall 2010). Enrichment per 10 kb bin was computed with the multiBigwigSummary tool in deepTools using the bins command. The outputs of multiBigwigSummary were *z*-scored and plotted in R with ggplot2 (version 3.5.0) (Wickham 2016).

### Statistics

Wilcoxon rank sum tests were run in R using the wilcox.test function. Permutation tests were also run in R, and as follows. Autosomes were collapsed, and the observed difference in mean loop size between the autosomes (A) and the X chromosomes (X) was calculated as our test statistic. Mean loop size was defined as the separation in base pairs at the local maxima of the log derivative of the P(s) (Goloborodko et al. 2016; Gassler et al. 2017; Polovnikov et al. 2023). Permutations were run (n = 10,000) by extracting slope values, and randomly sampling a chromosome (A or X) without replacement. For each permutation, the mean loop size of A and X was selected, and the difference was calculated. A distribution of differences was plotted with ggplot2 (version 3.5.0). We calculated the *P*-value as a proportion of permuted test statistics that were more extreme than the observed test statistic.

## Supplementary Material

msaf270_Supplementary_Data

## Data Availability

The datasets used and generated in this paper are provided in File S1. All data generated in this study were deposited to the Gene Expression Omnibus database under series numbers GSE267962 and GSE267963. The rescaffolded *P. pacificus* genome and lifted annotation of protein coding genes can be found at https://github.com/ercanlab/2024_Aharonoff_et_al.git. All scripts run in this study were deposited to the following GitHub repository: https://github.com/ercanlab/2024_Aharonoff_et_al.git.
