# Supplementary material for: Parallel Evolution of X Chromosome-Specific Structural Maintenance of Chromosomes Complexes in Two Nematode Lineages
Source: Mol Biol Evol. 2025 Oct 24;42(11):msaf270. doi: 10.1093/molbev/msaf270 (PMC12629237; doi:10.1093/molbev/msaf270)
Supplement: msaf270_Supplementary_Data [file msaf270_supplementary_data.zip › #6_Supplementary_figures.pdf]

FIGURE S1

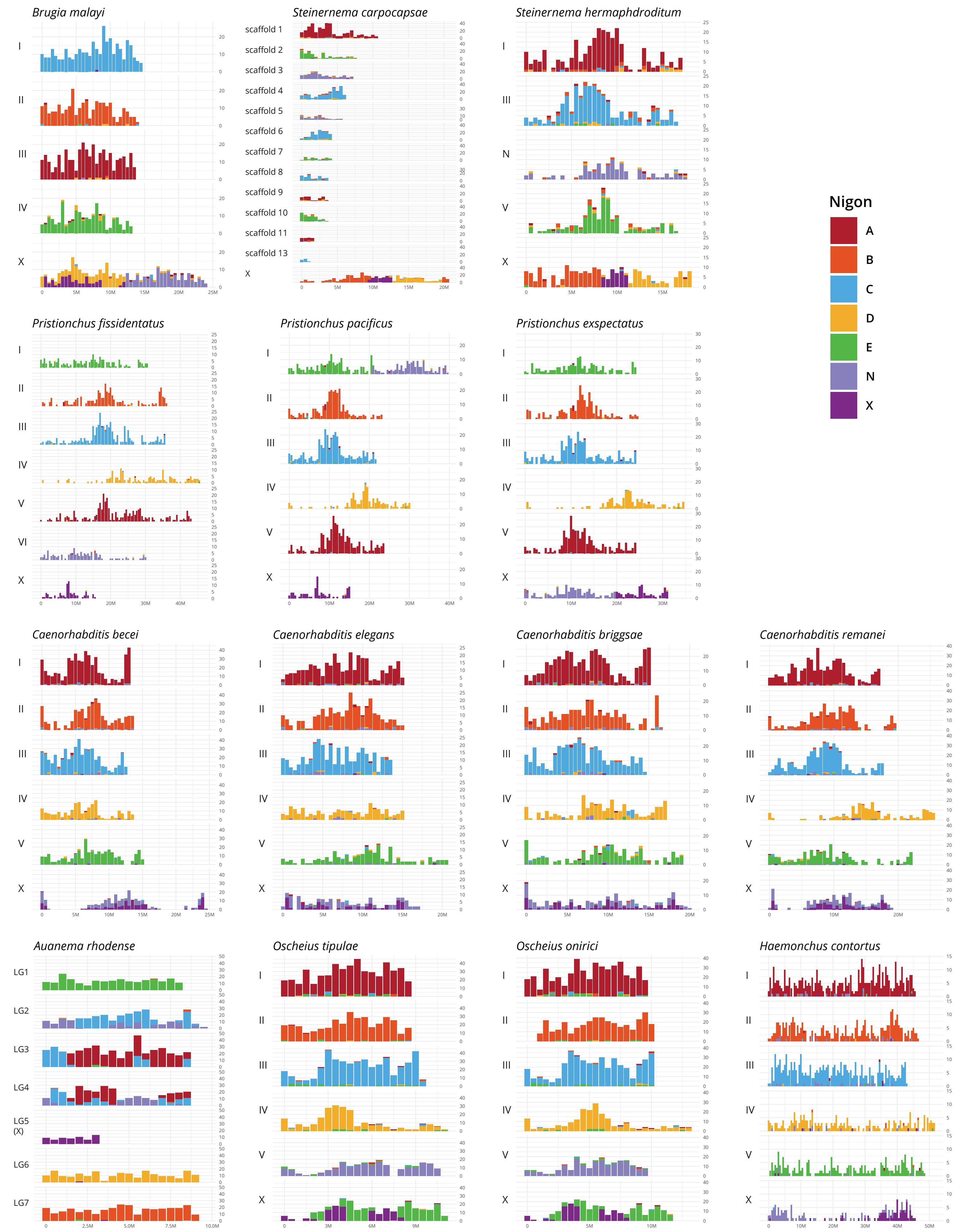

Brugia malayi
I
II
III
IV
X
Steinernema carpocapsae
scaffold 1
scaffold 2
scaffold 3
scaffold 4
scaffold 5
scaffold 6
scaffold 7
scaffold 8
scaffold 9
scaffold 10
scaffold 11
scaffold 13
X
Steinernema hermaphroditum
I
III
N
V
X
Pristionchus fissidentatus
I
II
III
IV
V
VI
X
Pristionchus pacificus
I
II
III
IV
V
X
Pristionchus exspectatus
I
II
III
IV
V
X
Caenorhabditis becei
I
II
III
IV
V
X
Caenorhabditis elegans
I
II
III
IV
V
X
Caenorhabditis briggsae
I
II
III
IV
V
X
Caenorhabditis remanei
I
II
III
IV
V
X
Auanema rhodense
LG1
LG2
LG3
LG4
LG5 (X)
LG6
LG7
Oscheius tipulae
I
II
III
IV
V
X
Oscheius onirici
I
II
III
IV
V
X
Haemonchus contortus
I
II
III
IV
V
X
Nigon
A
B
C
D
E
N
X

FIGURE S2

Gene tree, SDC-1

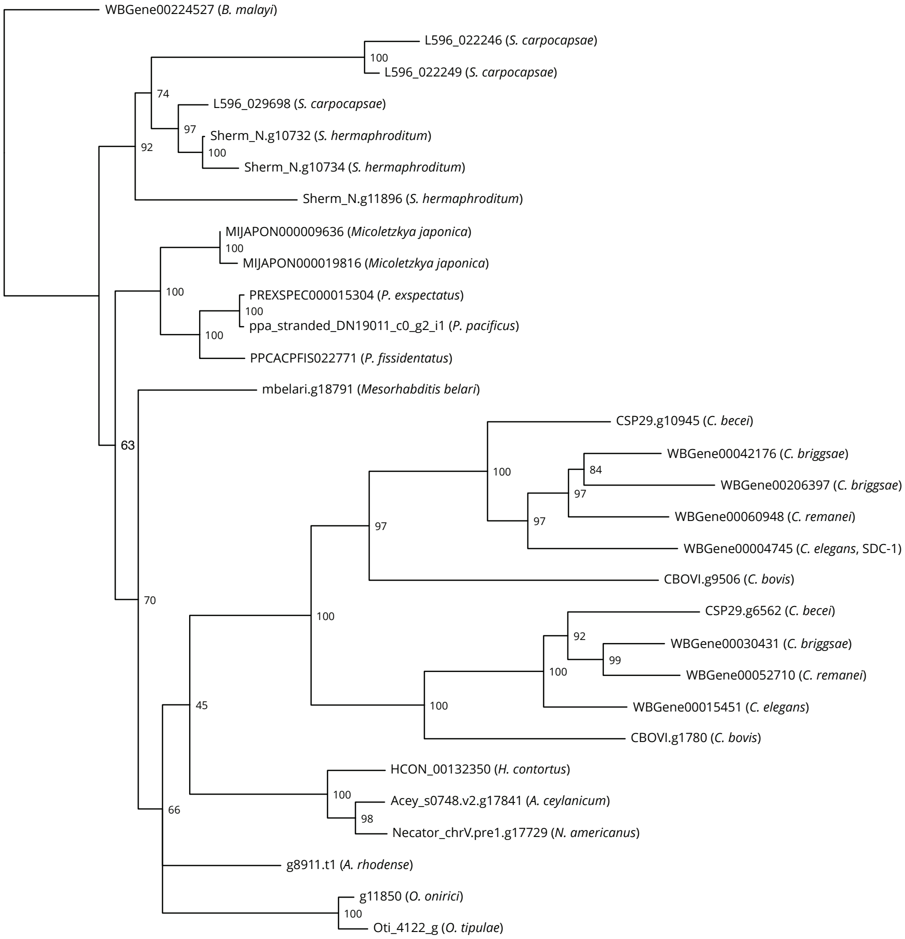

FIGURE S3

A

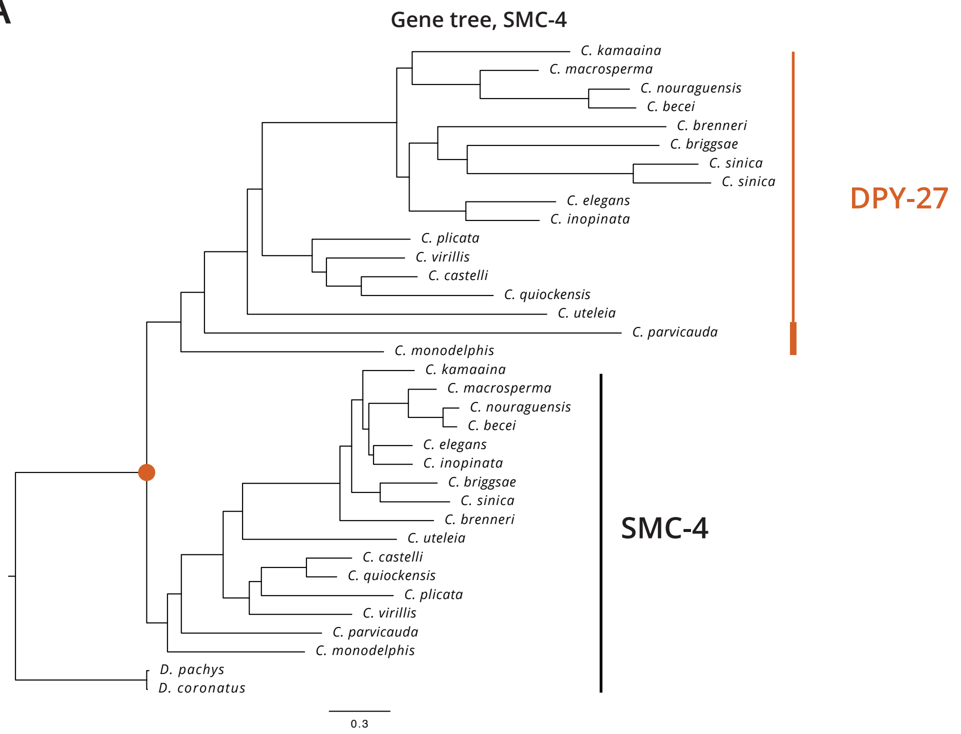

Gene tree, SMC-4
C. kamaaina
C. macrosperma
C. nouraguensis
C. becei
C. brenneri
C. briggsae
C. sinica
C. sinica
C. elegans
C. inopinata
C. plicata
C. virillis
C. castelli
C. quiockensis
C. uteleia
C. parvicauda
C. monodelphis
DPY-27
C. kamaaina
C. macrosperma
C. nouraguensis
C. becei
C. elegans
C. inopinata
C. briggsae
C. sinica
C. brenneri
C. uteleia
C. castelli
C. quiockensis
C. plicata
C. virillis
C. parvicauda
C. monodelphis
SMC-4
D. pachys
D. coronatus
0.3

B

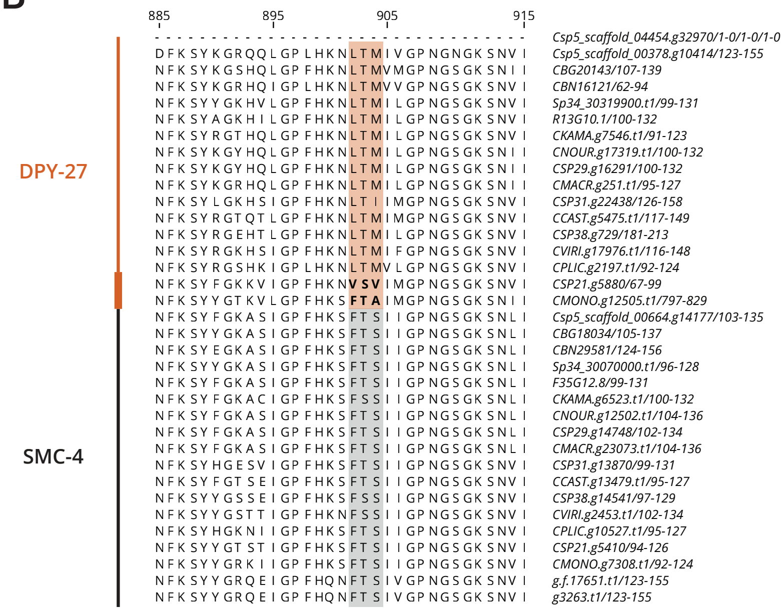

885
895
905
915
DPY-27
SMC-4
--------------------------------- Csp5_scaffold_04454.g32970/1-0/1-0/1-0
DFKSYKGRQQLGPLHKNLTMIVGPNGNGKSNVI Csp5_scaffold_00378.g10414/123-155
NFKSYKGSHQLGPFHKNLTMVMGPNGSGKSNII CBG20143/107-139
NFKSYKGRHQIGPLHKNLTMVVGPNGSGKSNVI CBN16121/62-94
NFKSYYGKHVLGPFHKNLTMILGPNGSGKSNVI Sp34_30319900.t1/99-131
NFKSYAGKHILGPFHKNLTMILGPNGSGKSNVI R13G10.1/100-132
NFKSYRGTHQLGPFHKNLTMILGPNGSGKSNVI CKAMA.g7546.t1/91-123
NFKSYKGYHQLGPFHKNLTMILGPNGSGKSNII CNOUR.g17319.t1/100-132
NFKSYKGYHQLGPFHKNLTMILGPNGSGKSNII CSP29.g16291/100-132
NFKSYKGRHQLGPFHKNLTMILGPNGSGKSNII CMACR.g251.t1/95-127
NFKSYLGKHSIGPFHKNLTIIMGPNGSGKSNVI CSP31.g22438/126-158
NFKSYRGTQTLGPFHKNLTMIMGPNGSGKSNVI CCAST.g5475.t1/117-149
NFKSYRGEHTLGPFHKNLTMILGPNGSGKSNVI CSP38.g729/181-213
NFKSYRGKHSIGPFHKNLTMIFGPNGSGKSNVI CVIRI.g17976.t1/116-148
NFKSYRGSHKIGPLHKNLTMVLGPNGSGKSNVI CPLIC.g2197.t1/92-124
NFKSYFGKKVIGPFHKNVSVIMGPNGSGKSNVI CSP21.g5880/67-99
NFKSYYGTKVLGPFHKSFTAIMGPNGSGKSNII CMONO.g12505.t1/797-829
NFKSYFGKASIGPFHKSFTSIIGPNGSGKSNLI Csp5_scaffold_00664.g14177/103-135
NFKSYYGKASIGPFHKSFTSIIGPNGSGKSNLI CBG18034/105-137
NFKSYEGKASIGPFHKSFTSIIGPNGSGKSNLI CBN29581/124-156
NFKSYYGKASIGPFHKSFTSIIGPNGSGKSNLI Sp34_30070000.t1/96-128
NFKSYFGKASIGPFHKSFTSIIGPNGSGKSNLI F35G12.8/99-131
NFKSYFGKACIGPFHKSFSSIIGPNGSGKSNLI CKAMA.g6523.t1/100-132
NFKSYFGKASIGPFHKSFTSIIGPNGSGKSNLI CNOUR.g12502.t1/104-136
NFKSYFGKASIGPFHKSFTSIIGPNGSGKSNLI CSP29.g14748/102-134
NFKSYFGKASIGPFHKSFTSIIGPNGSGKSNLI CMACR.g23073.t1/104-136
NFKSYHGESVIGPFHKSFTSIIGPNGSGKSNVI CSP31.g13870/99-131
NFKSYFGTSEIGPFHKSFTSIIGPNGSGKSNVI CCAST.g13479.t1/95-127
NFKSYYGSSEIGPFHKSFSSIIGPNGSGKSNVI CSP38.g14541/97-129
NFKSYYGSTTIGPFHKNFSSIIGPNGSGKSNVI CVIRI.g2453.t1/102-134
NFKSYHGKNIIGPFHKSFTSIIGPNGSGKSNVI CPLIC.g10527.t1/95-127
NFKSYYGTSTIGPFHKSFTSIIGPNGSGKSNVI CSP21.g5410/94-126
NFKSYYGRKIIGPFHKSFTSIIGPNGSGKSNVI CMONO.g7308.t1/92-124
NFKSYYGRQEIGPFHQNFTSIVGPNGSGKSNVI g.f.17651.t1/123-155
NFKSYYGRQEIGPFHQNFTSIVGPNGSGKSNVI g3263.t1/123-155

FIGURE S4

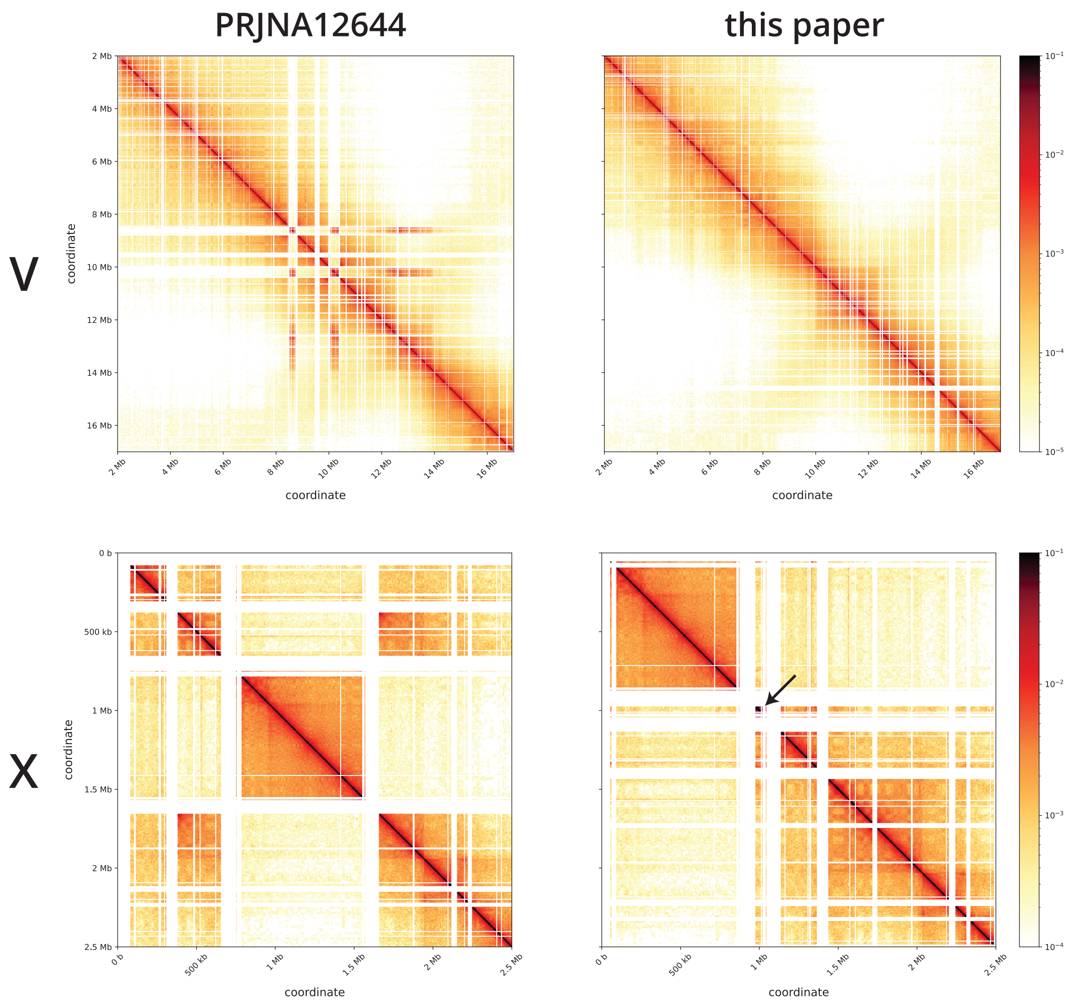

PRJNA12644
this paper
V
X
coordinate
2 Mb
4 Mb
6 Mb
8 Mb
10 Mb
12 Mb
14 Mb
16 Mb
0 b
500 kb
1 Mb
1.5 Mb
2 Mb
2.5 Mb

FIGURE S5

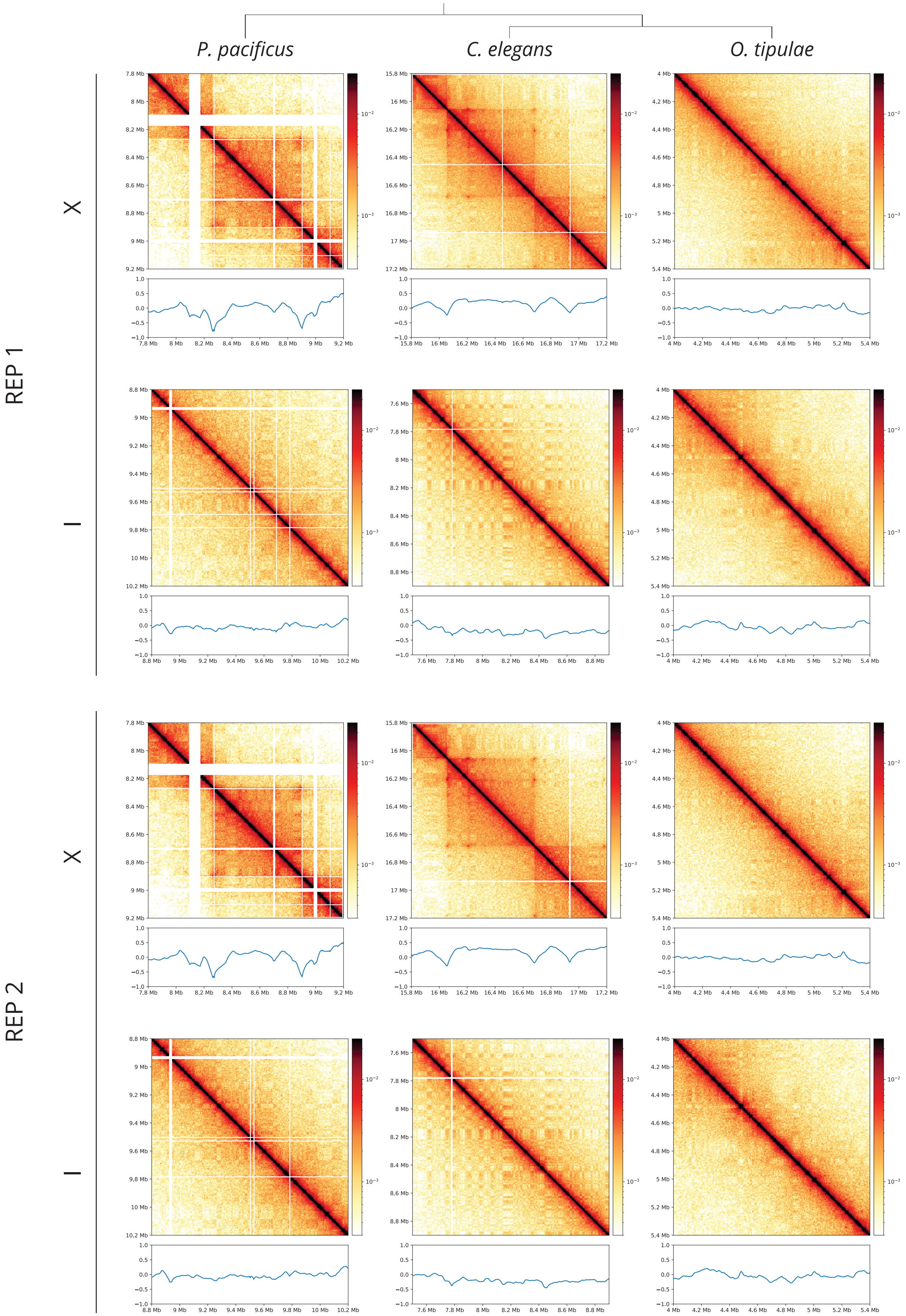

FIGURE S6

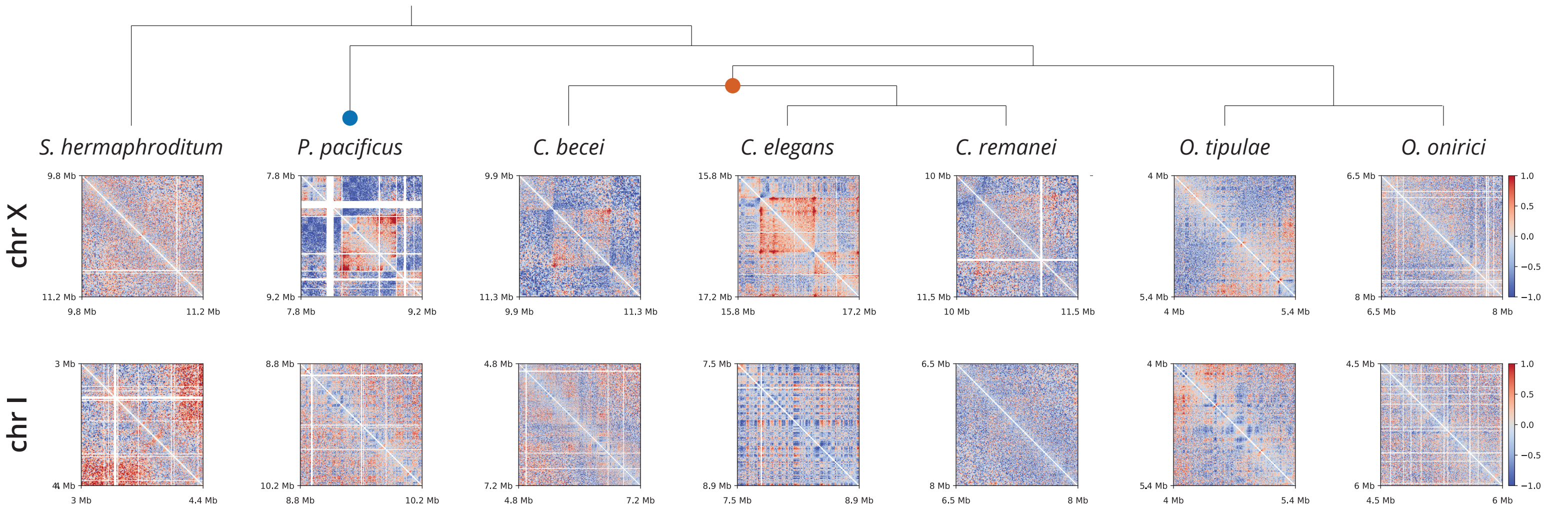
S. hermaphroditum
P. pacificus
C. becei
C. elegans
C. remanei
O. tipulae
O. onirici
chr X
chr I
9.8 Mb
11.2 Mb
7.8 Mb
9.2 Mb
9.9 Mb
11.3 Mb
15.8 Mb
17.2 Mb
10 Mb
11.5 Mb
4 Mb
5.4 Mb
6.5 Mb
8 Mb
3 Mb
4.4 Mb
8.8 Mb
10.2 Mb
4.8 Mb
7.2 Mb
7.5 Mb
8.9 Mb
4.5 Mb
6 Mb
1.0
0.5
0.0
−0.5
−1.0

FIGURE S7

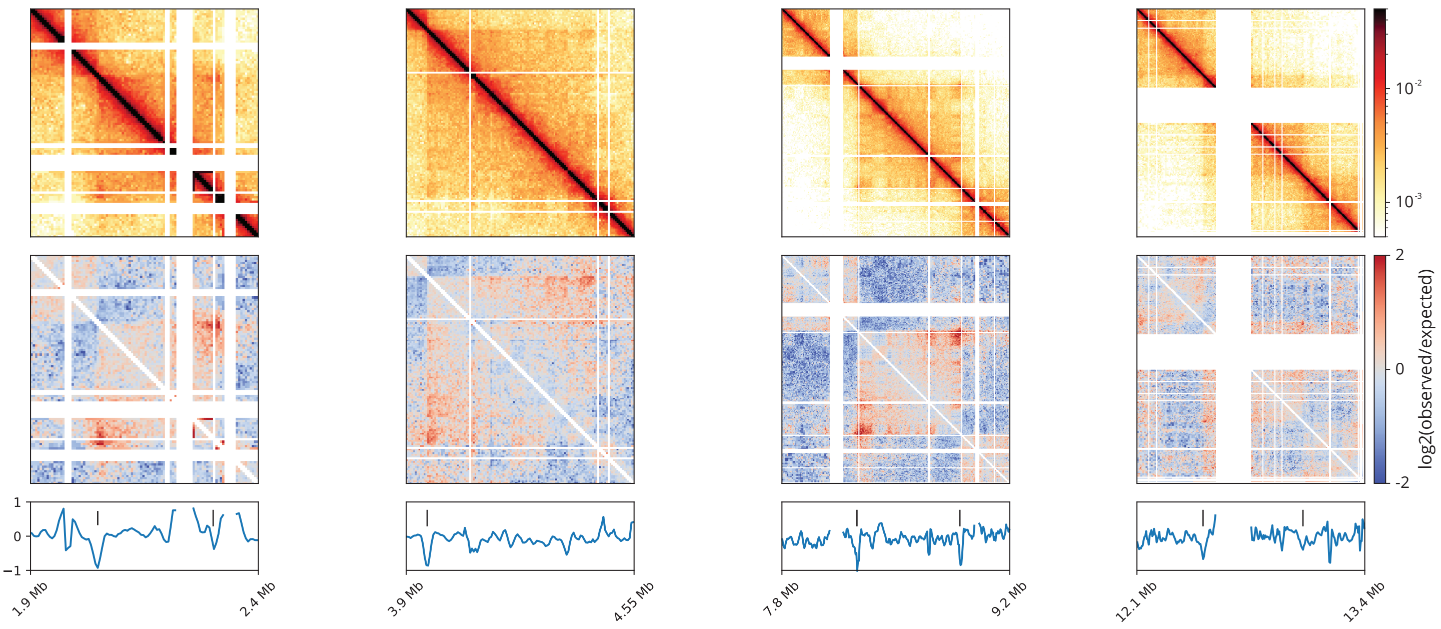

10^-2
10^-3
2
0
-2
log2(observed/expected)
1
0
−1
1.9 Mb
2.4 Mb
3.9 Mb
4.55 Mb
7.8 Mb
9.2 Mb
12.1 Mb
13.4 Mb

FIGURE S8

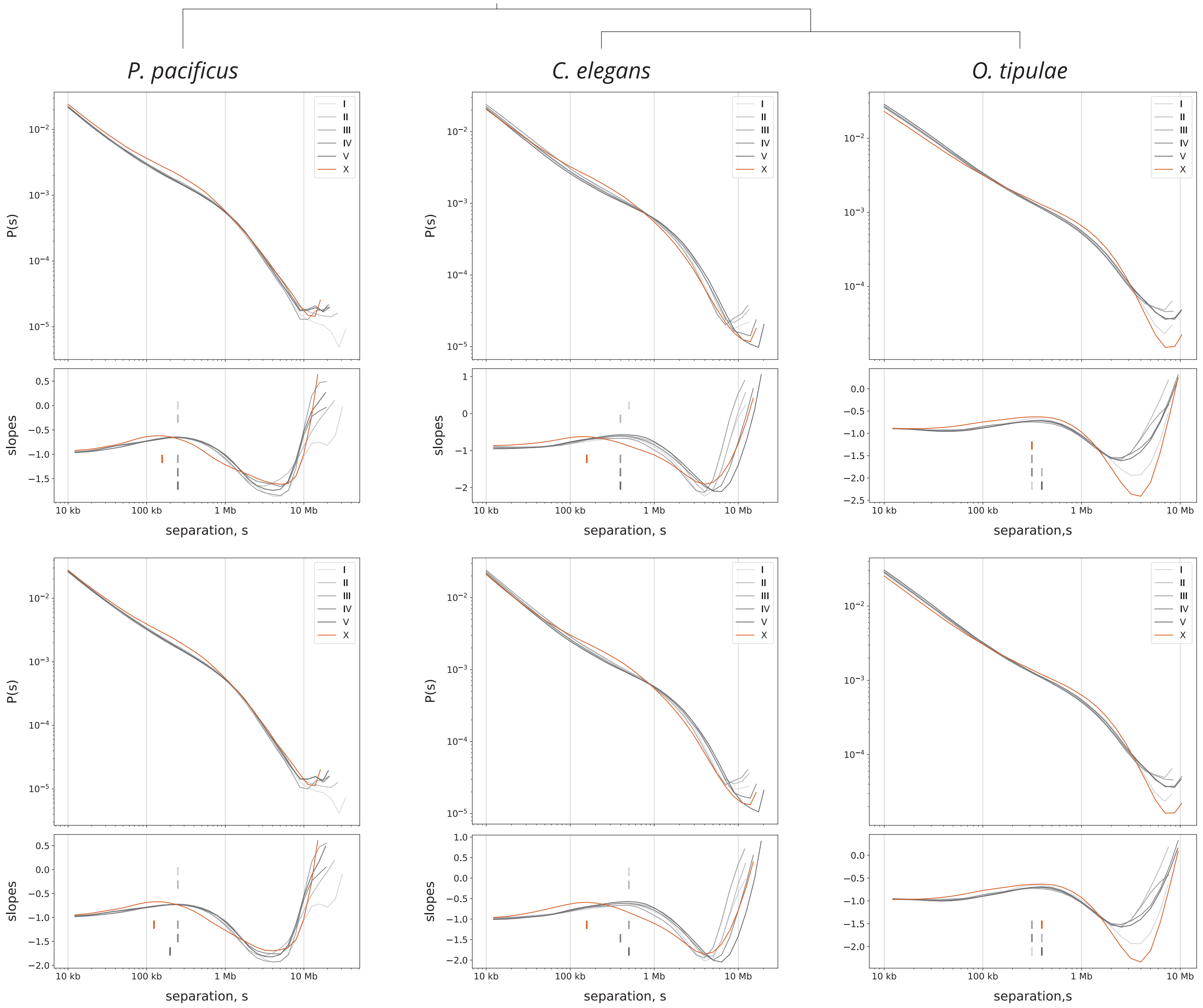
P. pacificus
C. elegans
O. tipulae
P(s)
slopes
separation, s
separation,s
I
II
III
IV
V
X
10 kb
100 kb
1 Mb
10 Mb

FIGURE S9

Permutation analysis of shifted average loop size

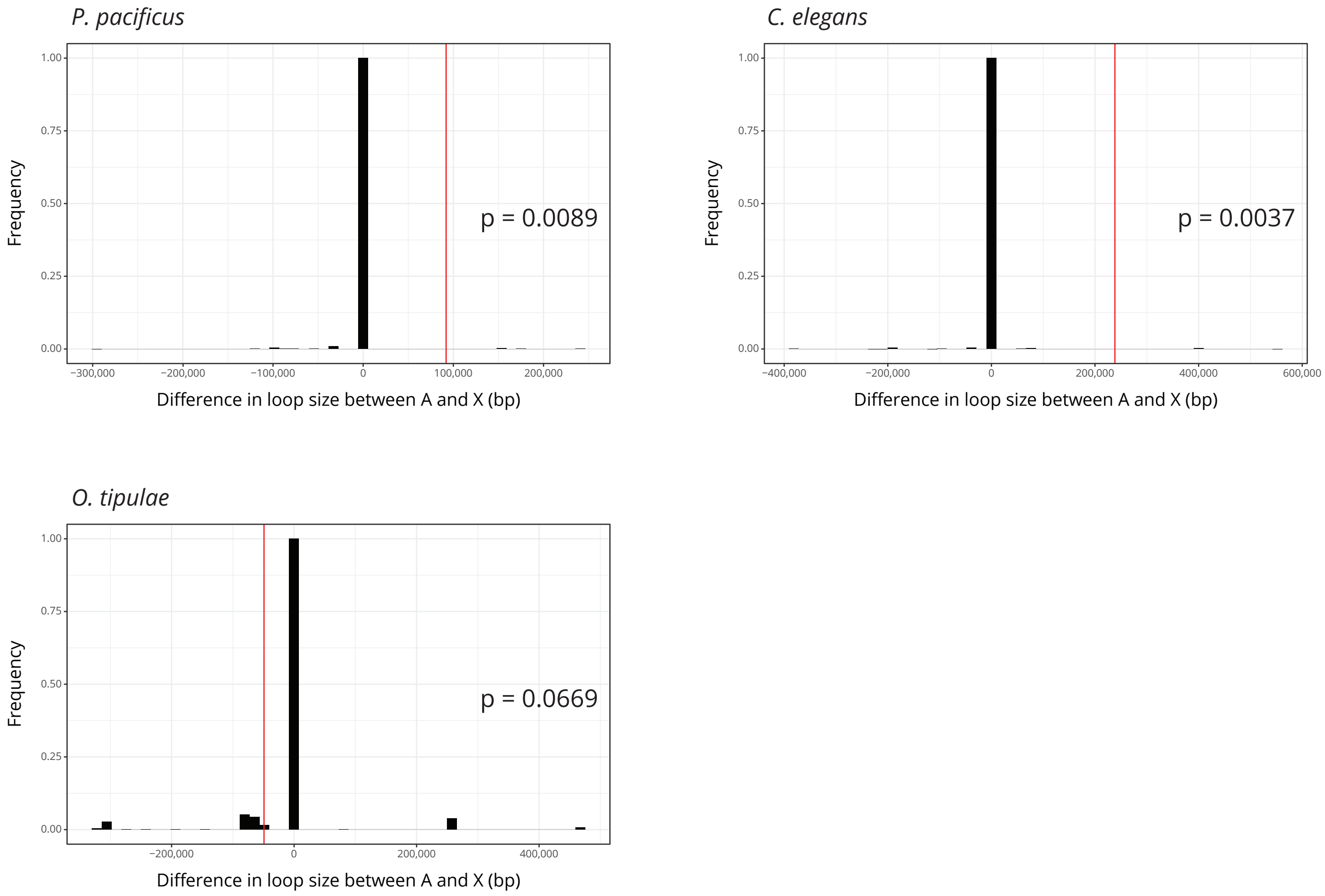

FIGURE S10

MIX-I

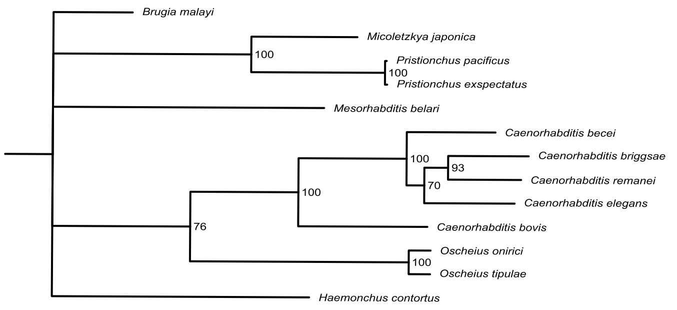

DPY-26

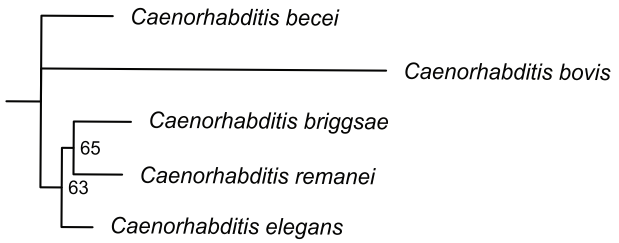

DPY-28

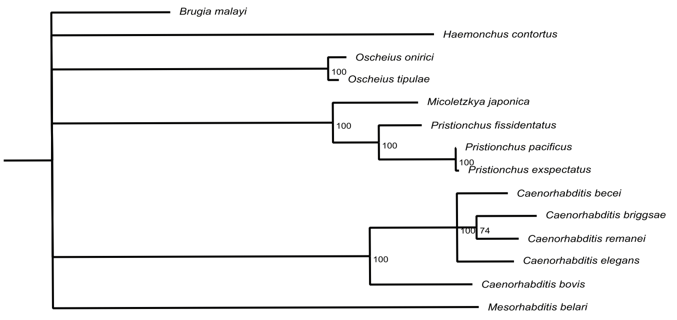

CAPG-1

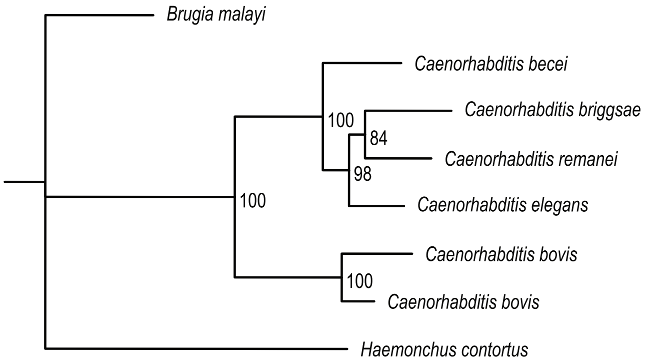

HCP-6

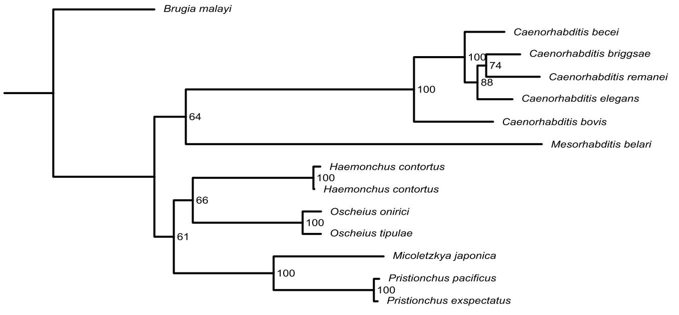

KLE-2

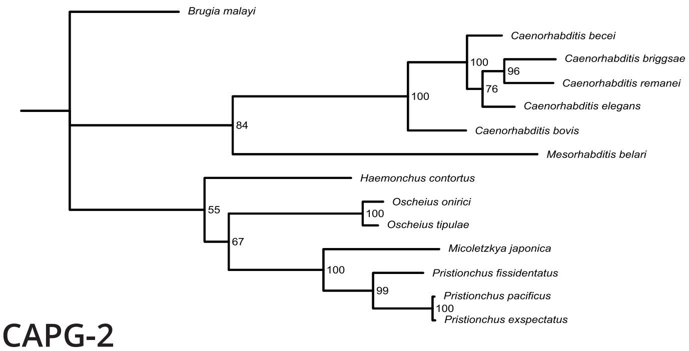

CAPG-2

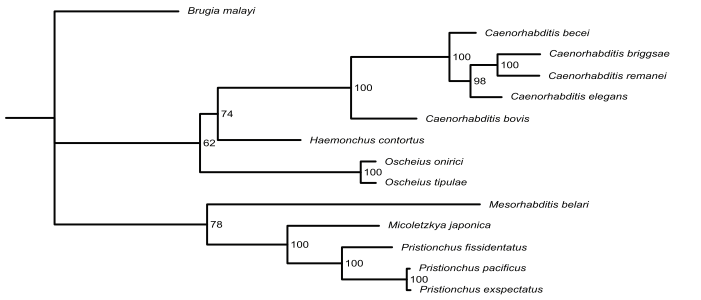

SMC-5

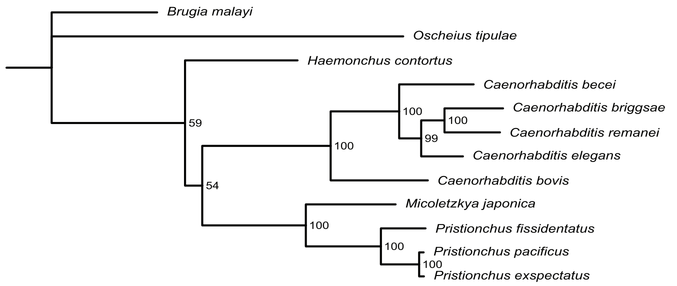

SMC-6

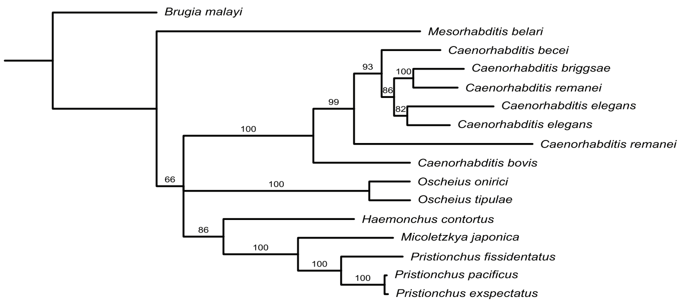

SMC-1

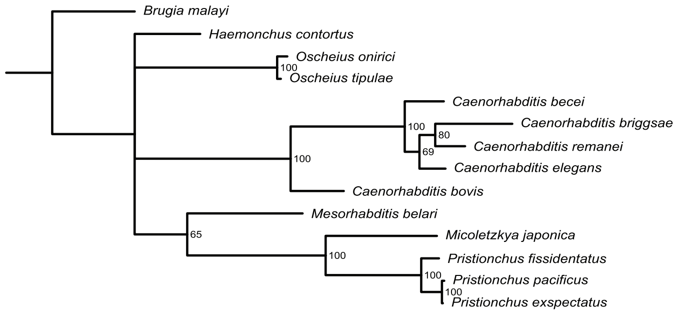

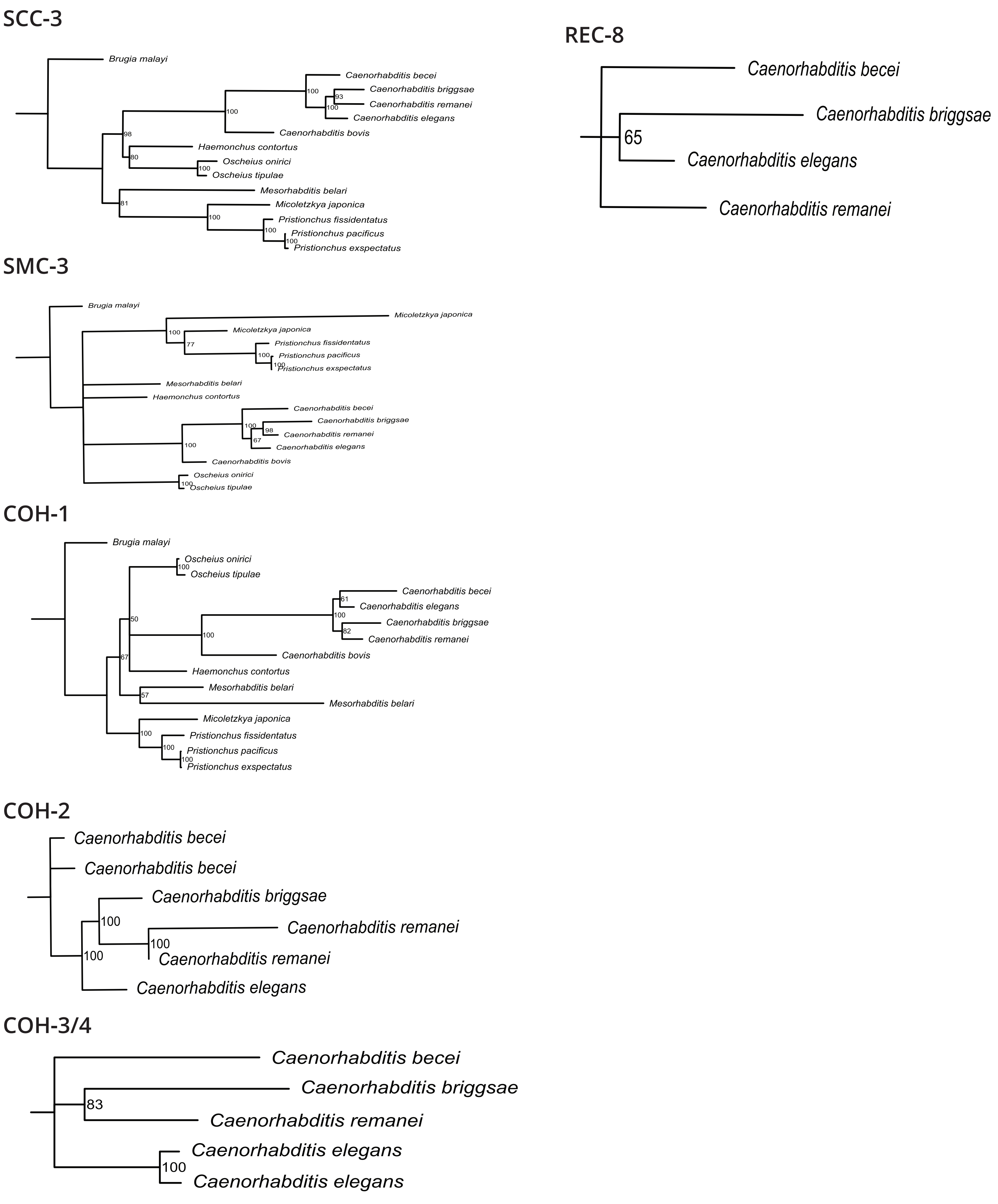
SCC-3
Brugia malayi
Caenorhabditis becei
Caenorhabditis briggsae
Caenorhabditis remanei
Caenorhabditis elegans
Caenorhabditis bovis
Haemonchus contortus
Oscheius onirici
Oscheius tipulae
Mesorhabditis belari
Micoletzkya japonica
Pristionchus fissidentatus
Pristionchus pacificus
Pristionchus exspectatus
100
93
100
100
98
80
100
81
100
100
100
SMC-3
Brugia malayi
Micoletzkya japonica
Micoletzkya japonica
Pristionchus fissidentatus
Pristionchus pacificus
Pristionchus exspectatus
Mesorhabditis belari
Haemonchus contortus
Caenorhabditis becei
Caenorhabditis briggsae
Caenorhabditis remanei
Caenorhabditis elegans
Caenorhabditis bovis
Oscheius onirici
Oscheius tipulae
100
77
100
100
100
98
67
100
100
COH-1
Brugia malayi
Oscheius onirici
Oscheius tipulae
Caenorhabditis becei
Caenorhabditis elegans
Caenorhabditis briggsae
Caenorhabditis remanei
Caenorhabditis bovis
Haemonchus contortus
Mesorhabditis belari
Mesorhabditis belari
Micoletzkya japonica
Pristionchus fissidentatus
Pristionchus pacificus
Pristionchus exspectatus
100
61
100
82
50
100
67
57
100
100
100
COH-2
Caenorhabditis becei
Caenorhabditis becei
Caenorhabditis briggsae
Caenorhabditis remanei
Caenorhabditis remanei
Caenorhabditis elegans
100
100
100
COH-3/4
Caenorhabditis becei
Caenorhabditis briggsae
Caenorhabditis remanei
Caenorhabditis elegans
Caenorhabditis elegans
83
100
REC-8
Caenorhabditis becei
Caenorhabditis briggsae
Caenorhabditis elegans
Caenorhabditis remanei
65

FIGURE S11

Distribution of |log2(hermaphrodite/male)|, mRNA-seq

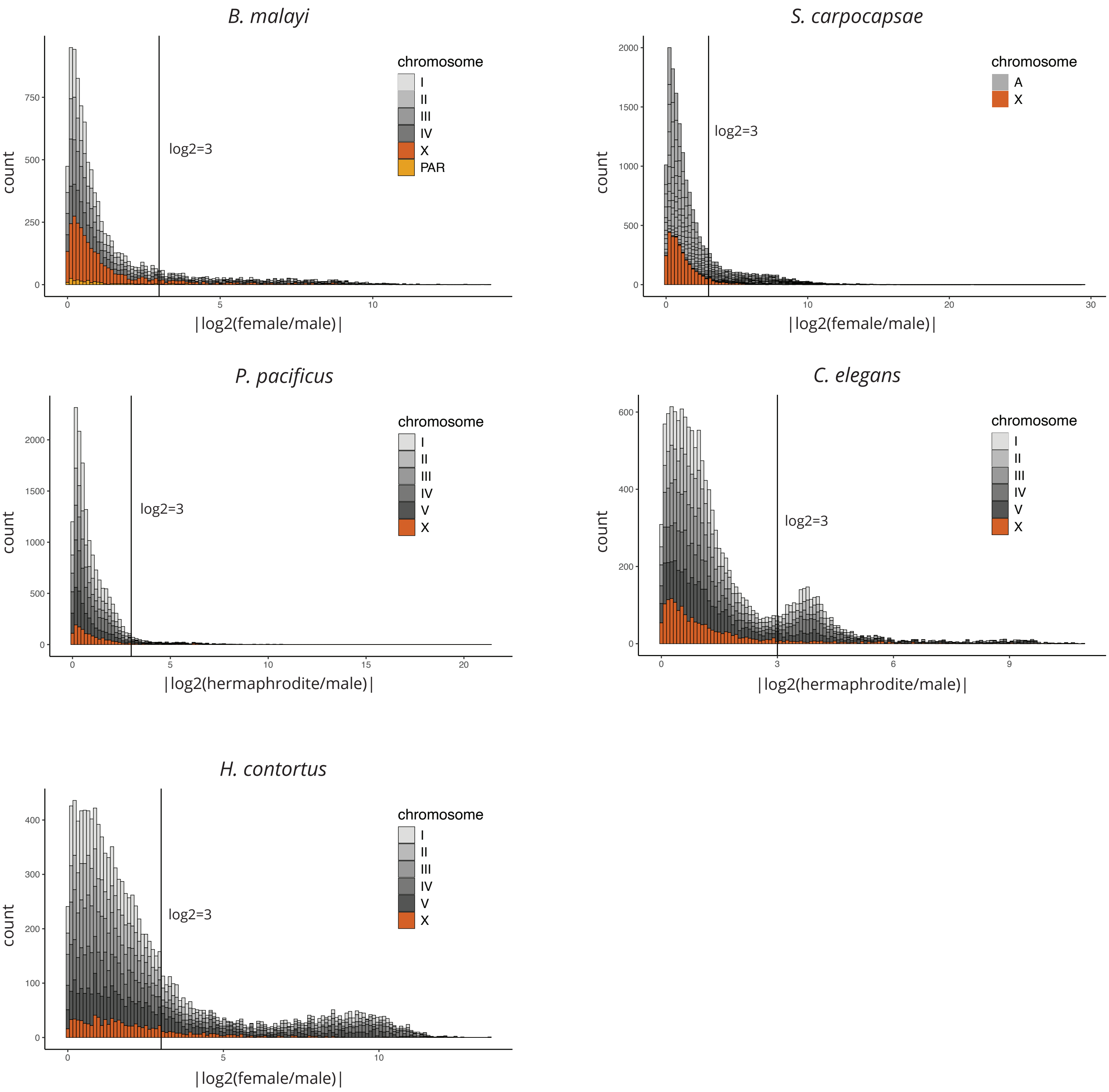

FIGURE S12

Differential expression between females/hermaphrodites and males, whole worm mRNA-seq, soma enriched genes

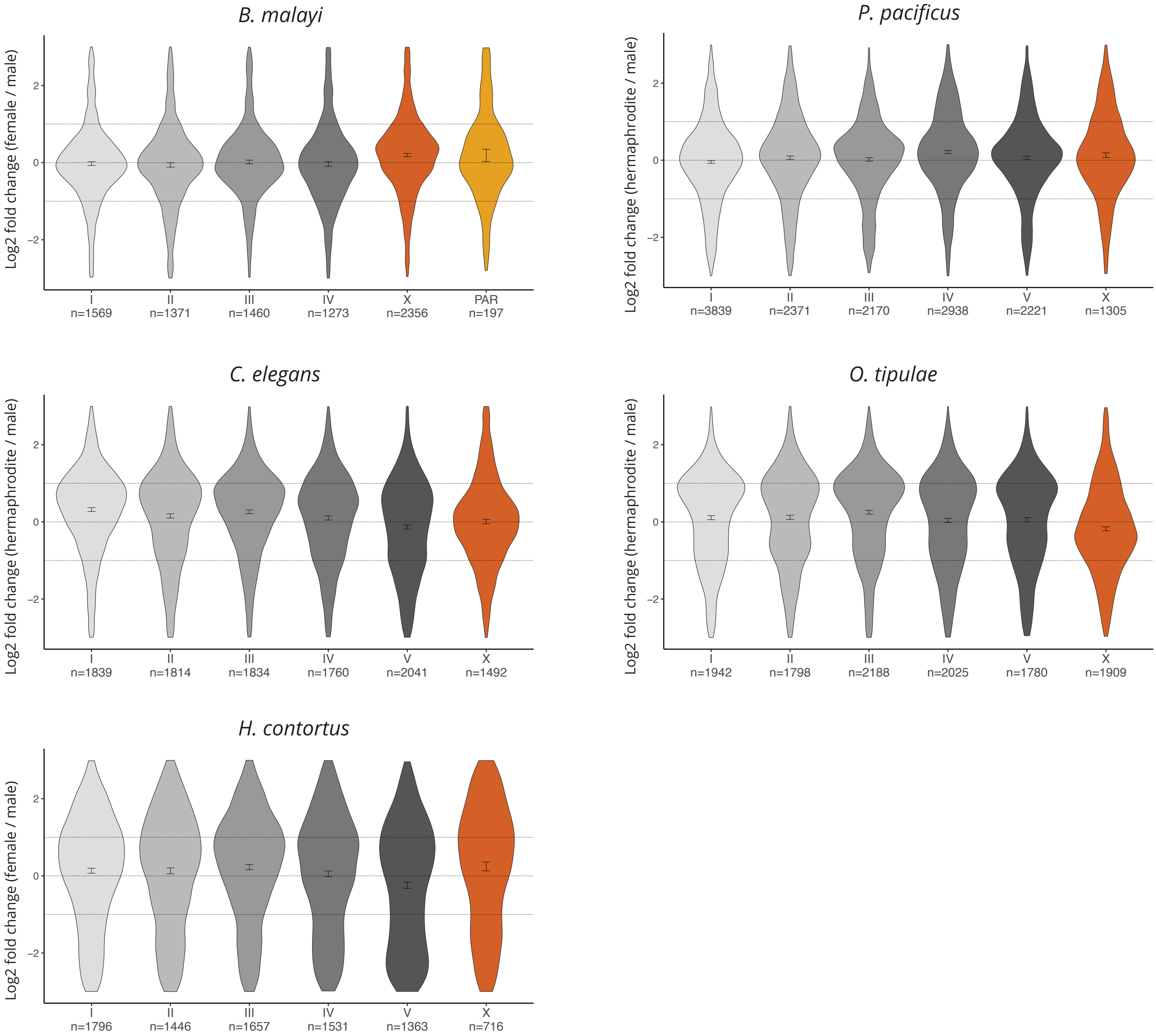

FIGURE S13

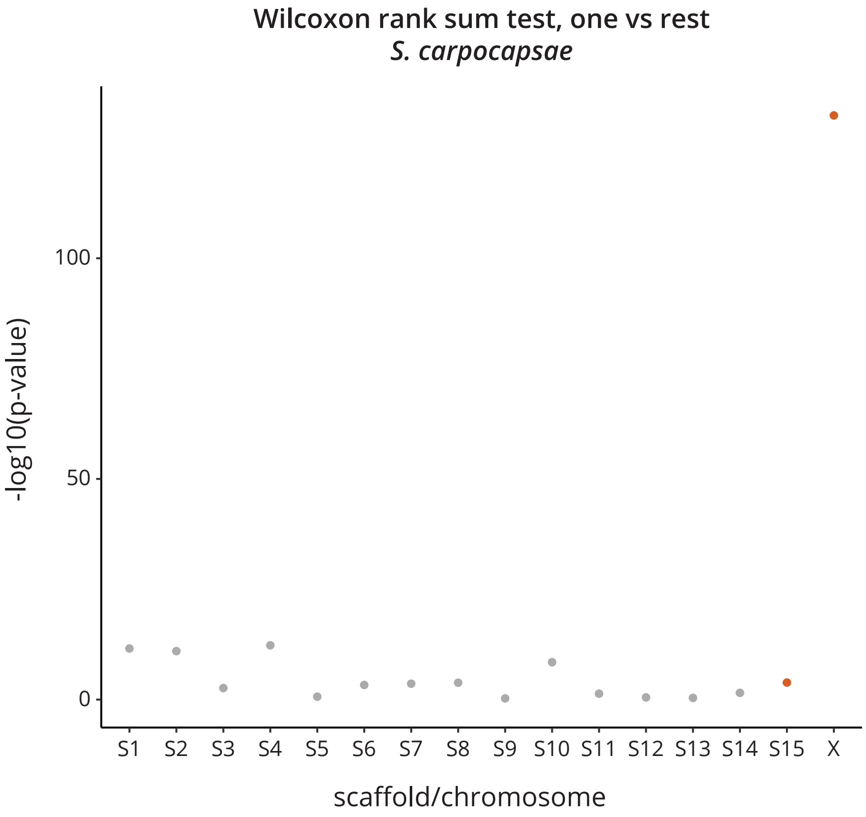

Wilcoxon rank sum test, one vs rest
S. carpocapsae
100
50
0
-log10(p-value)
S1
S2
S3
S4
S5
S6
S7
S8
S9
S10
S11
S12
S13
S14
S15
X
scaffold/chromosome

FIGURE S14

A

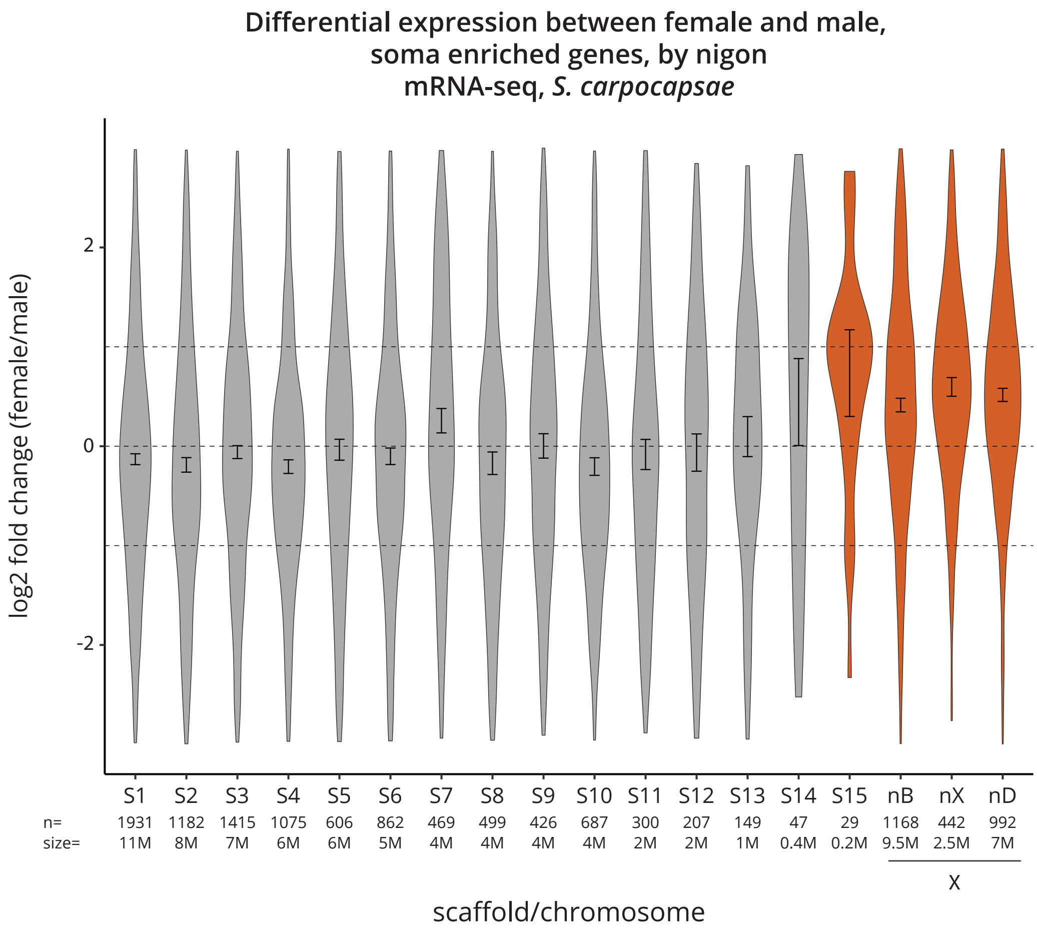

B

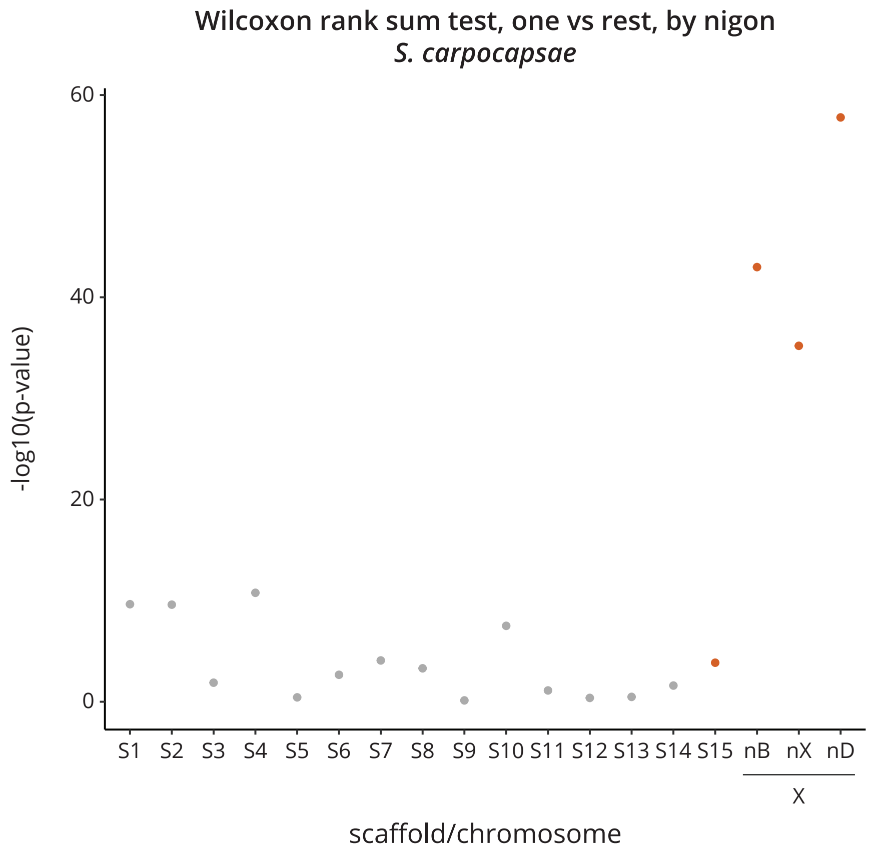

FIGURE S15

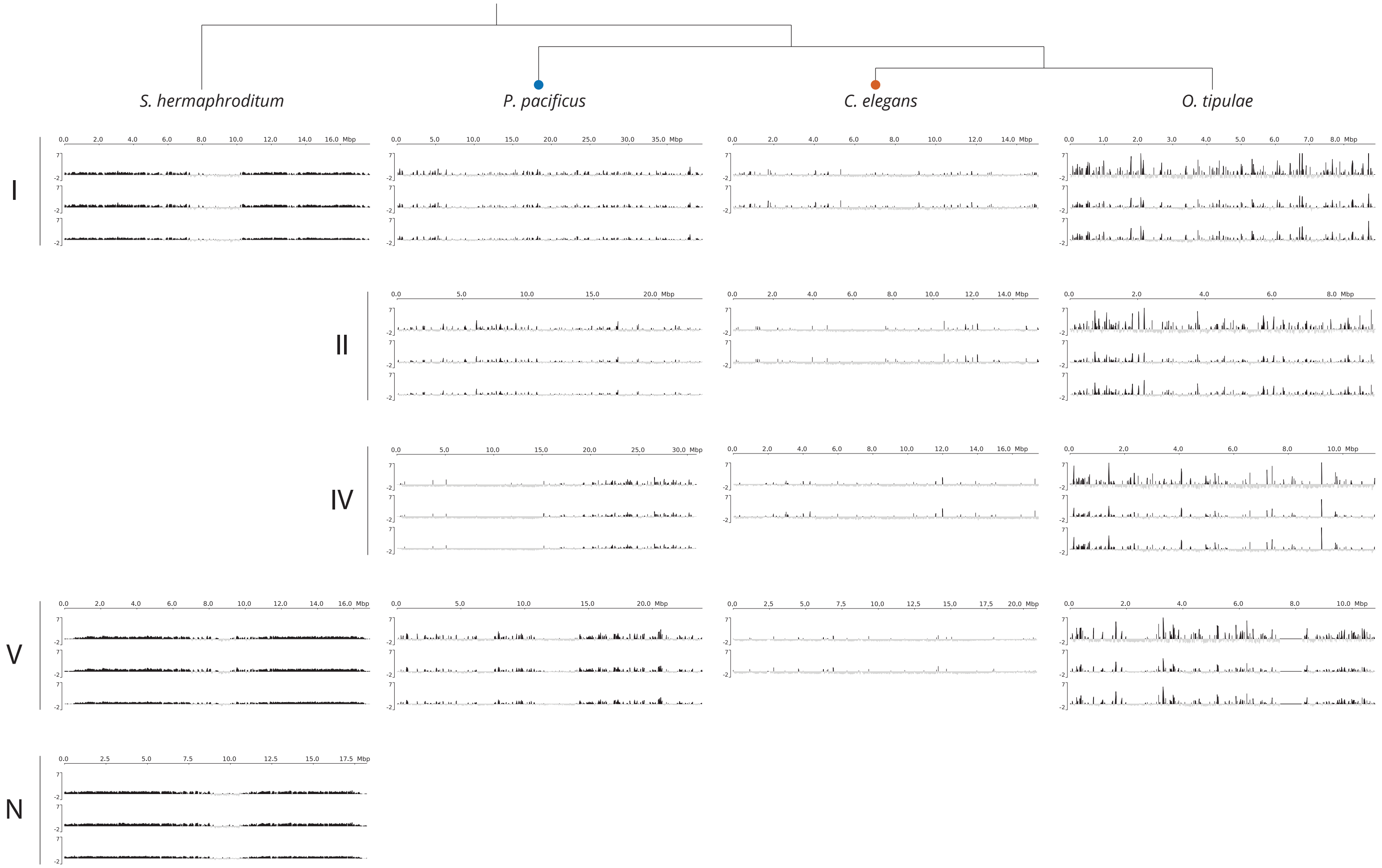

S. hermaphroditum
P. pacificus
C. elegans
O. tipulae
I
II
IV
V
N
Mbp

FIGURE S16

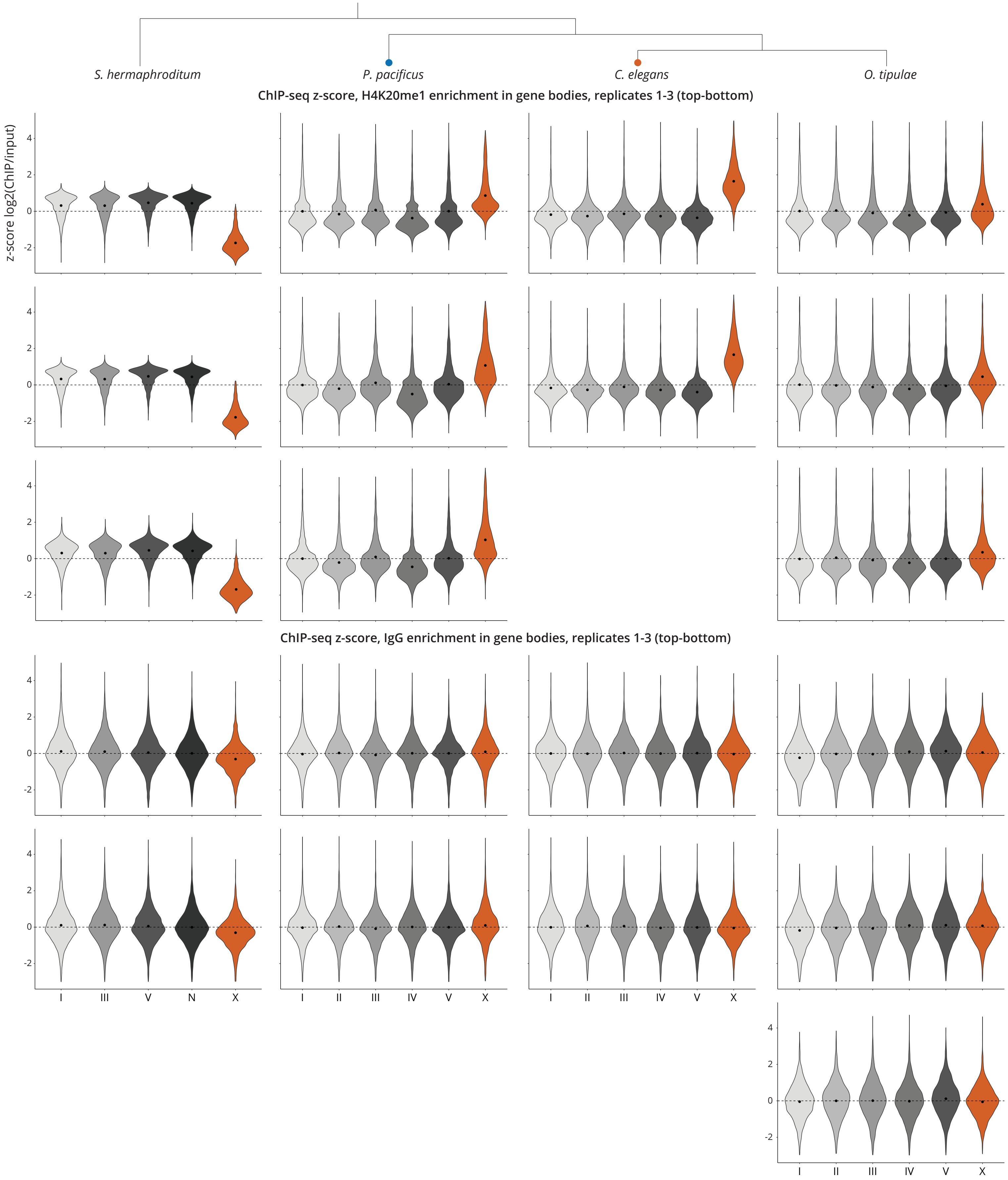

S. hermaphroditum
P. pacificus
C. elegans
O. tipulae
ChIP-seq z-score, H4K20me1 enrichment in gene bodies, replicates 1-3 (top-bottom)
z-score log2(ChIP/input)
ChIP-seq z-score, IgG enrichment in gene bodies, replicates 1-3 (top-bottom)
4
2
0
-2
I
III
V
N
X
I
II
III
IV
V
X

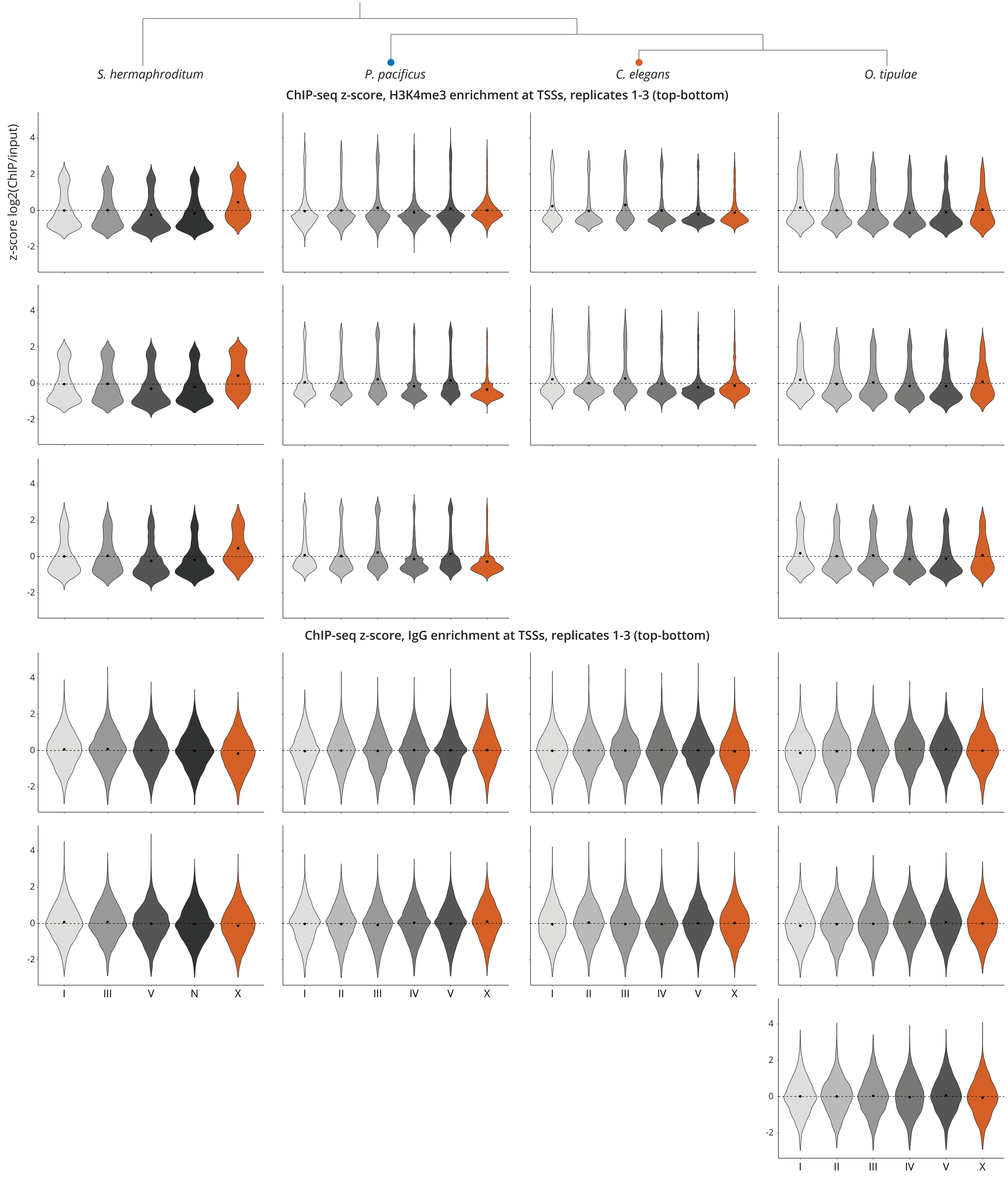
S. hermaphroditum
P. pacificus
C. elegans
O. tipulae
ChIP-seq z-score, H3K4me3 enrichment at TSSs, replicates 1-3 (top-bottom)
z-score log2(ChIP/input)
ChIP-seq z-score, IgG enrichment at TSSs, replicates 1-3 (top-bottom)
I III V N X
I II III IV V X
I II III IV V X
I II III IV V X

FIGURE S17

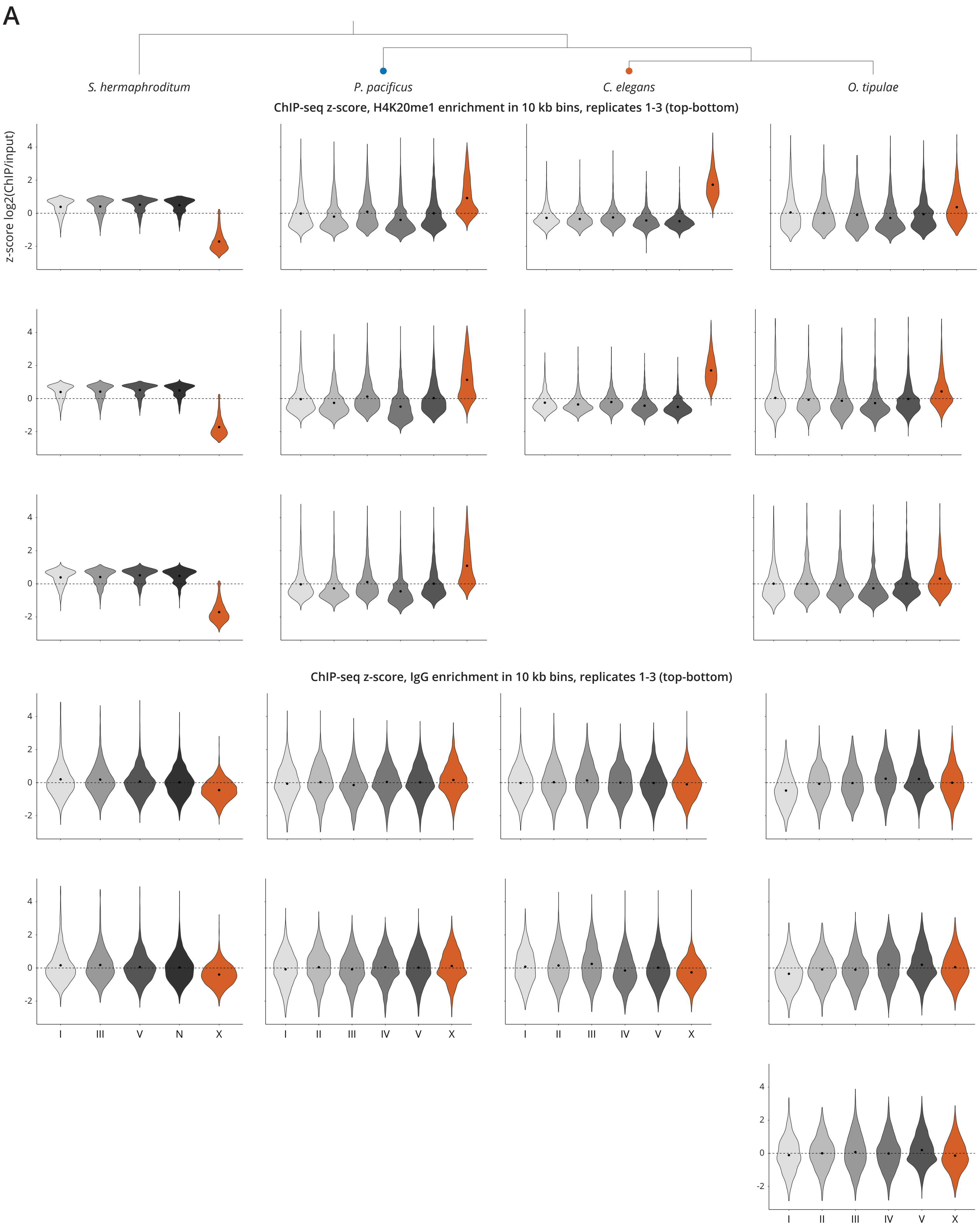
A
S. hermaphroditum
P. pacificus
C. elegans
O. tipulae
ChIP-seq z-score, H4K20me1 enrichment in 10 kb bins, replicates 1-3 (top-bottom)
z-score log2(ChIP/input)
4
2
0
-2
ChIP-seq z-score, IgG enrichment in 10 kb bins, replicates 1-3 (top-bottom)
I
III
V
N
X
I
II
III
IV
V
X

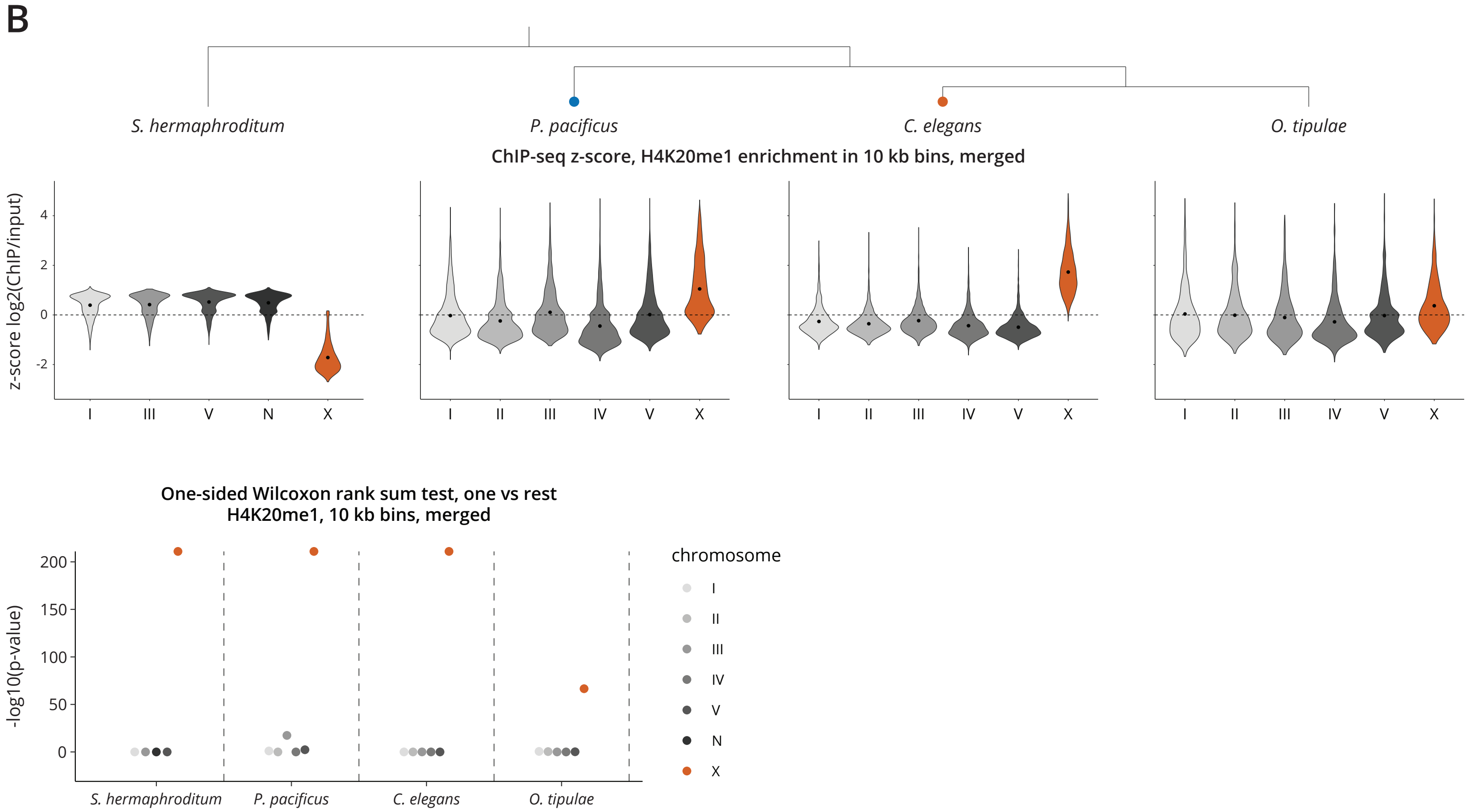

B
S. hermaphroditum
P. pacificus
C. elegans
O. tipulae
ChIP-seq z-score, H4K20me1 enrichment in 10 kb bins, merged
z-score log2(ChIP/input)
4
2
0
-2
I
II
III
IV
V
N
X
One-sided Wilcoxon rank sum test, one vs rest
H4K20me1, 10 kb bins, merged
-log10(p-value)
200
150
100
50
0
chromosome

FIGURE S18

A

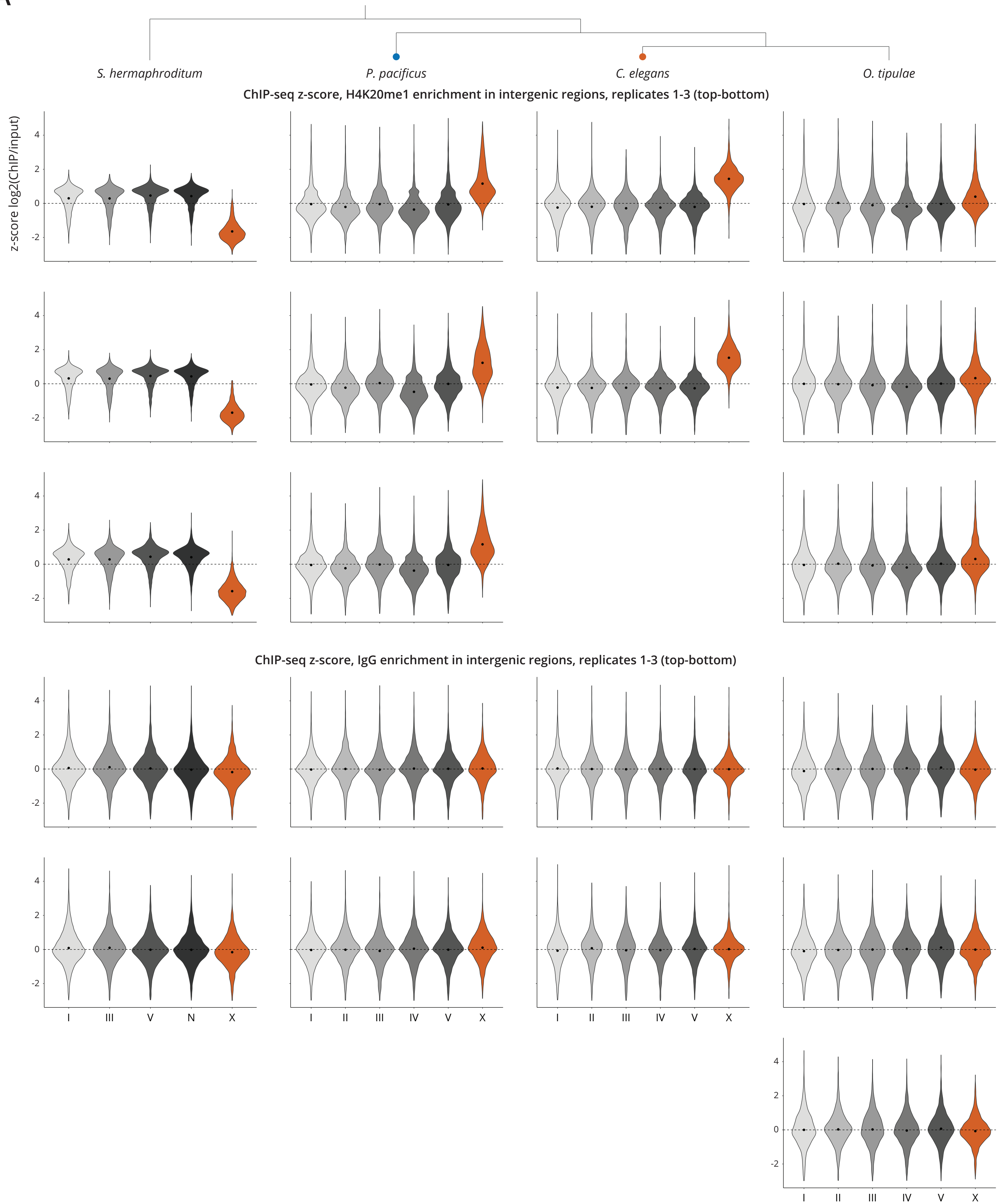
S. hermaphroditum
P. pacificus
C. elegans
O. tipulae
ChIP-seq z-score, H4K20me1 enrichment in intergenic regions, replicates 1-3 (top-bottom)
z-score log2(ChIP/input)
4
2
0
-2
ChIP-seq z-score, IgG enrichment in intergenic regions, replicates 1-3 (top-bottom)
I
III
V
N
X
I
II
III
IV
V
X

B

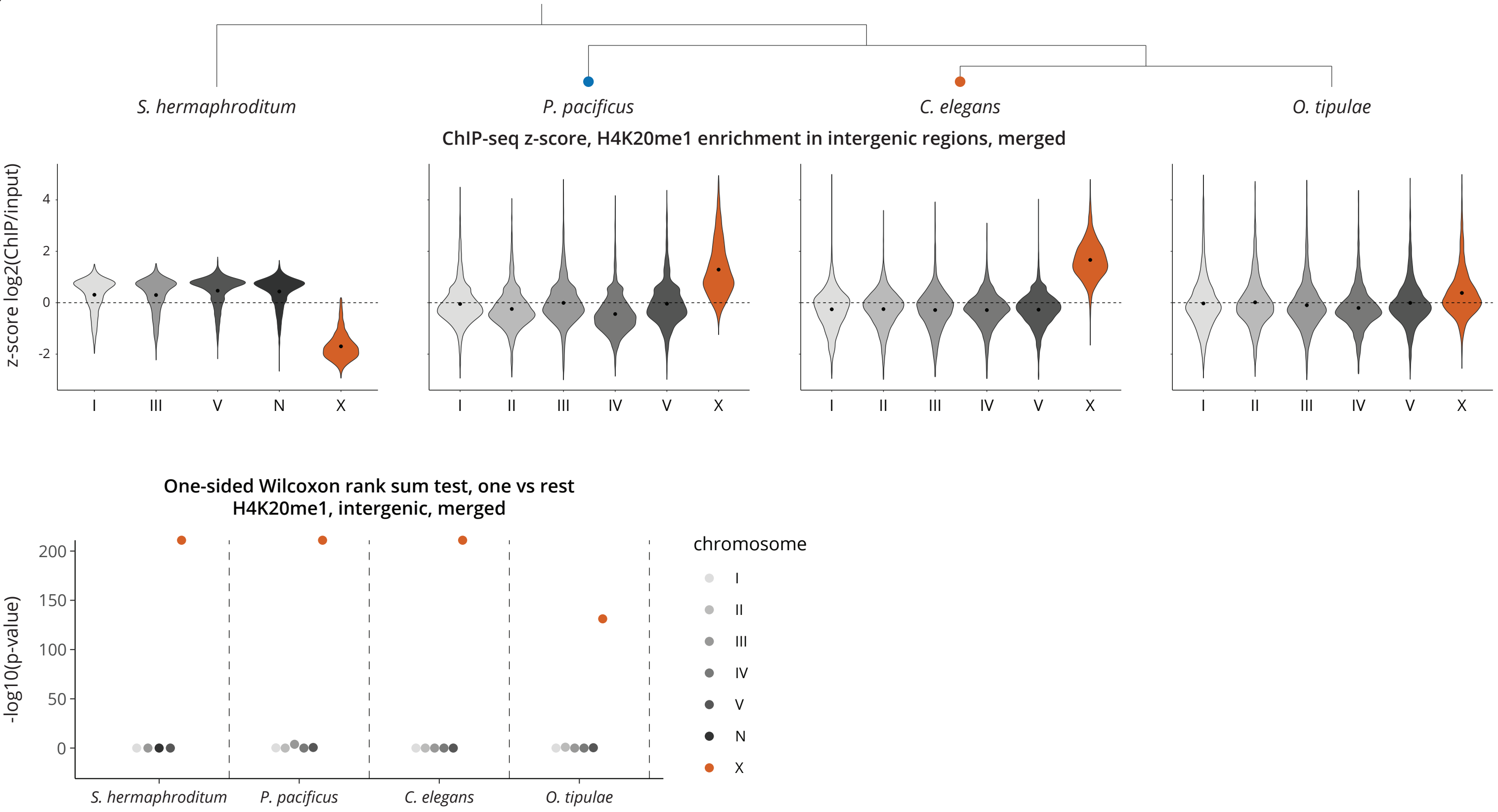
S. hermaphroditum
P. pacificus
C. elegans
O. tipulae
ChIP-seq z-score, H4K20me1 enrichment in intergenic regions, merged
z-score log2(ChIP/input)
4
2
0
-2
I
III
V
N
X
I
II
III
IV
V
X
One-sided Wilcoxon rank sum test, one vs rest
H4K20me1, intergenic, merged
-log10(p-value)
200
150
100
50
0
S. hermaphroditum
P. pacificus
C. elegans
O. tipulae
chromosome
I
II
III
IV
V
N
X

FIGURE S19

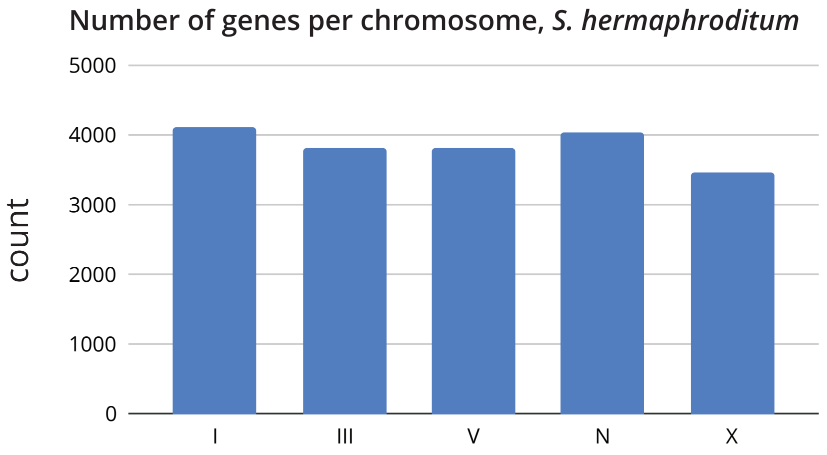

Number of genes per chromosome, *S. hermaphroditum*
count
5000
4000
3000
2000
1000
0
I
III
V
N
X

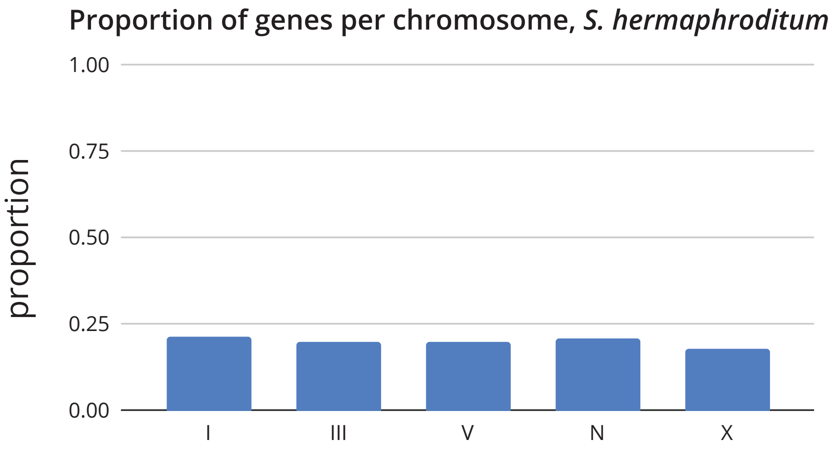

Proportion of genes per chromosome, *S. hermaphroditum*
proportion
1.00
0.75
0.50
0.25
0.00
I
III
V
N
X
