# Supplementary material for: Parallel Evolution of X Chromosome-Specific Structural Maintenance of Chromosomes Complexes in Two Nematode Lineages
Source: Mol Biol Evol. 2025 Oct 24;42(11):msaf270. doi: 10.1093/molbev/msaf270 (PMC12629237; doi:10.1093/molbev/msaf270)
Supplement: msaf270_Supplementary_Data [file msaf270_supplementary_data.zip › #6_Supplementary_figure_legends.pdf]

Figure S1. Nigon painted chromosomes of sampled nematodes. For each species, each chromosome or scaffold is represented as a subgraph that shows the number of loci mapped to a nonoverlapping bin (500 kb) in the form of a stacked histogram colored by ancestral Nigon element. The x and y axes of each subgraph, which represent chromosome length from start to end and number of loci, respectively, are scaled to the maxima for each species. Chromosomes painted by ancestral Nigon elements for species in Spirurina, Tylenchina, and Rhabditina point to multiple independent autosome to X chromosome fusions in nematodes, and significant rearrangements of the nematode X chromosome.

Figure S2. Gene tree of SDC-1. A maximum-likelihood gene tree of SDC-1 was made with the OrthoFinder results in Spirurina, Tylenchina, and Rhabditina. SDC-1 is conserved in these three clades, and expanded a few times through partial duplications. Node values reflect bootstrap scores.

Figure S3. Gene tree of SMC-4 in *Caenorhaditis*. (A) OrthoFinder was run on a group of sixteen *Caenorhabiditis* species with *D. pachys* and *D. coronatus* as outgroups (see **supplemental file 1**), and a maximum-likelihood gene tree for SMC-4 was made. SMC-4 duplication occurred before the earliest known branch in *Caenorhabditis*, *C. monodelphis*, and after the split between *Caenorhabditis* and *Diploscapter* (orange circle). The scale bar and node values reflect branch length and bootstrap scores, respectively. (B) Multiple sequence alignment of the N-terminal ATPase domain shows that the LTM motif is exclusive to DPY-27 and is conserved after the *C. parvicauda* split. Tick marked numbers reflect coordinate in the multiple sequence alignment. Numbers by gene names reflect coordinate in sequence.

Figure S4. Fixed misjoins and inversions in the *P. pacificus* genome. Misjoins and inversions (left panel) on chromosome V and X of the *P. pacificus* genome were fixed (right panel) with Juicebox Assembly Tools, and pbcontig517 was placed on the left arm of chromosome X (arrow) using the scaffolding tool YaHS (Zhou et al. 2023). X and y axes represent chromosome coordinates, and colorbar reflects relative interaction between a pair of bins, shown on a logarithmic scale.

Figure S5. Replicates of Hi-C matrices and insulation scores. Replicates of Hi-C matrices (5 kb resolution) and insulation scores (150 kb window size) for data generated in this study (*P. pacificus* and *O. tipulae*) exhibit reproducibility in displaying X chromosome specific TADs. *C. elegans* N2 data is from our previously published paper (Kim et al. 2022).

Figure S6. Observed/expected plots for Hi-C matrices. Observed/expected plots were computed by calculating the expected contact frequency for each matrix, and dividing the observed over the expected matrix. In the Hi-C data from mixed sex, mixed stage samples, observed/expected plots allow us to observe the otherwise concealed loop anchors, e.g. in *C. remanei*. X and y axes reflect chromosome coordinates, and the color bar reflects log2(observed/expected).

Figure S7. Hi-C matrices of the observed X chromosome TADs in *P. pacificus*. Hi-C matrices of each of the four observed TADs on the *P. pacificus* X chromosome (top panel) with log2(observed/expected) plots (middle panel). Dips in insulation score (bottom panel, 20 kb window size) represent TAD boundaries and are marked by ticks. Empty bins (white) resulting in insulation score dips were not considered.

Figure S8. Replicates of distance decay curves. Replicates of P(s) curve and its derivative exhibit reproducibility of estimated mean loop size.

Figure S9. The observed difference in mean loop size between the autosomes and X chromosome are significantly different in *P. pacificus* and *C. elegans*. Permutations were performed by randomly permuting the slope values between the X chromosome and autosomes, extracting the corresponding separation in bp, and subtracting the X from the A as our test statistic. The distribution represents n=10000 permutations. Red line, observed difference; p, proportion of permutations that were more extreme than the observed difference.

Figure S10. Maximum-likelihood trees of all known SMC proteins in nematodes. Maximum likelihood trees were generated for all condensin, cohesin, and SMC-5/6 subunits, which show no other SMC duplications in *P. pacificus*. See **supplemental file 1** for list of species used.

Figure S11. Distribution of |log2 fold change| in *B. malayi*, *S. carpocapsae*, *P. pacificus*, *C. elegans*, and *H. contortus*. The distribution of differentially expressed genes between sexes is split into soma (log2 < 3) and sex-biased, germline enriched genes (log2 > 3). The dearth of germline enriched genes in *P. pacificus* was addressed in Albritton et al. (2014).

Figure S12. Log2 fold change plots separated by chromosome. Log2 fold change between female (or hermaphrodite) and males of soma enriched expressed genes in *B. malayi*, *P. pacificus*, *C. elegans*, *O. tipulae*, and *H. contortus* show little to no difference between chromosomes. PAR, pseudoautosomal region.

Figure S13. *S. carpocapsae* X chromosomes display significant differential expression when compared to autosomes. -log10(p-value) from two-sided Wilcoxon rank sum tests comparing the mean log2 fold change of a scaffold/chromosome to the mean of the rest (e.g., mean of X and mean of S1-14). Here, the differential expression observed between sexes on the X chromosome cannot be explained by the existing variation in autosomes between sexes. Scaffold 15 is a supposed piece of the X chromosome with only 29 genes (less than 0.01% of the genes on the X chromosome) (Serra et al. 2019).

Figure S14. Each Nigon element on the *S. carpocapsae* X chromosome is incompletely dosage compensated. (A) The X chromosome of *S. carpocapsae* was split by its Nigon elements (see **Figure S1**) and the log2 fold difference in expression between females and males was plotted as a function of scaffold/chromosome. The three Nigon elements, B, X and D, display between 1.3-1.5 times more expression in females than males, which is consistent with incomplete dosage compensation. n represents the number of genes, and the size of each

chromosome is shown in megabases, M. (B) -log10(p-value) from two-sided Wilcoxon rank sum tests comparing the mean log2 fold change of a scaffold/chromosome to the mean of the rest (e.g., mean of X and mean of S1-14) with the X chromosome split by Nigon element. nB = Nigon element B; nX = Nigon element X; nD = Nigon element D.

Figure S15. Whole chromosome H4K20me1 ChIP-seq tracks for autosomes. H4K20me1 ChIP-seq enrichment tracks (ChIP minus input) are shown for chromosomes I, II, IV, V, and N in *S. hermaphroditum*, *P. pacificus*, *C. elegans*, and *O. tipulae* hermaphrodite larvae. Two to three biological replicates are shown per species. Negative values on each track are due to normalization to input data and are faded.

Figure S16. Replicates of enrichment of H4K20me1 at gene bodies and H3K4me3 at TSSs. Replicates of ChIP-seq z-score analysis exhibit reproducibility. IgG is shown as a negative control.

Figure S17. H4K20me1 is enriched along the X chromosome of *P. pacificus* and *O. tipulae*. (A) Chromosomes were binned (10 kb) and enrichment was measured per bin. H4K20me1 enrichment (z-scored log2(ChIP/input)) was plotted for each chromosome. H4K20me1 is consistently enriched across biological replicates along the *P. pacificus* and *O. tipulae* X chromosomes, but is depleted from the *S. hermaphroditum* X chromosome. IgG is shown as a negative control. (B) The replicates were merged, and enrichment is shown for each species. (C) -log10(p-value) from one-sided Wilcoxon rank sum tests comparing the mean log2(ChIP/input) of a chromosome to the mean of the rest (e.g., mean of X and mean of I-V). Statistics were run on the merged data.

Figure S18. H4K20me1 is enriched in the intergenic regions on the X chromosomes of *P. pacificus* and *O. tipulae*. (A) H4K20me1 ChIP enrichment in intergenic regions (z-scored log2(ChIP/input)) was plotted for each chromosome. H4K20me1 is consistently enriched in intergenic regions across biological replicates on the *P. pacificus* and *O. tipulae* X chromosomes, but is depleted on the *S. hermaphroditum* X chromosome. (B) Replicates were merged and enrichment is shown for each species. (C) -log10(p-value) from Wilcoxon rank sum tests comparing the mean log2(ChIP/input) of a chromosome to the mean of the rest (e.g., mean of X and mean of I-V). Statistics were run on the merged data.

Figure S19. **Distribution of genes on the *S. hermaphroditum* chromosomes.** The number (top) and proportion (bottom) of genes per chromosome were plotted. Genes in *S. hermaphroditum* are distributed similarly among its five chromosomes, thus depletion of H4K20me1 on the X chromosomes is not due to lack of genes.

REFERENCES

Kim J, Jimenez DS, Ragipani B, Zhang B, Street LA, Kramer M, Albritton SE, Winterkorn LH, Morao AK, Ercan S. 2022. Condensin DC loads and spreads from recruitment sites to create loop-anchored TADs in C. elegans. *Elife* 11:e68745.

Serra L, Macchietto M, Macias-Muñoz A, McGill CJ, Rodriguez IM, Rodriguez B, Murad R, Mortazavi A. 2019. Hybrid Assembly of the Genome of the Entomopathogenic Nematode Steinernema carpocapsae Identifies the X-Chromosome. *G3 (Bethesda)* 9:2687–2697.

Zhou C, McCarthy SA, Durbin R. 2023. YaHS: yet another Hi-C scaffolding tool. *Bioinformatics* 39:btac808.
